# Effect of Early High-Dose Recombinant Human Erythropoietin on Behavior and Quality of Life in Children Aged 5 Years Born Very Preterm: Secondary Analysis of a Randomized Clinical Trial

**DOI:** 10.1001/jamanetworkopen.2022.45499

**Published:** 2022-12-07

**Authors:** Eleonora Picotti, Tilman Reinelt, Brigitte Koller, Hans Ulrich Bucher, Christoph M. Rüegger, Jean-Claude Fauchère, Giancarlo Natalucci

**Affiliations:** 1 Newborn Research, Department of Neonatology, University Hospital Zurich, University of Zurich, Zurich, Switzerland; 2 Larsson-Rosenquist Center for Neurodevelopment, Growth, and Nutrition of the Newborn, Department of Neonatology, University Hospital Zurich, University of Zurich, Zurich, Switzerland; 3 Child Development Center, University Children’s Hospital Zurich, Zurich, Switzerland

## Abstract

**Importance:**

In light of the promising neuroprotective properties of recombinant human erythropoietin (RHEpo), the Swiss EPO Neuroprotection Trial was started to investigate its effect on neurodevelopment in very preterm infants. The results of the primary and secondary outcome analysis did not show any effect of RHEpo on cognitive performance, neuromotor outcomes, or somatic growth of the study participants at ages 2 or 5 years.

**Objective:**

To investigate whether early high-dose RHEpo improves behavioral outcomes and health-related quality of life (HRQoL) at age 5 years.

**Design, Setting, and Participants:**

This was a prespecified secondary analysis of the double-blind, placebo-controlled, multicenter Swiss EPO Neuroprotection randomized clinical trial, which was conducted at 5 level-III perinatal centers in Switzerland. Infants born between 26 weeks 0 days’ and 31 weeks 6 days’ gestation were recruited between 2005 and 2012 and followed-up until age 5 years (last follow-up in 2018). Data were analyzed from January 6 to December 31, 2021.

**Interventions:**

Infants were assigned to receive either RHEpo (3000 IU/kg) or placebo (saline, 0.9%) intravenously 3 times within the first 42 hours after birth.

**Main Outcomes and Measures:**

The prespecified parent-reported measures of behavioral outcomes and health-related quality of life (HRQoL) of their children at the age of 5 years were assessed by two standardized questionnaires: the Strengths and Difficulties Questionnaire (behavioral outcomes) and the KIDSCREEN-27 (HRQoL).

**Results:**

Among 448 randomized infants, 228 infants were assigned to the RHEpo group and 220 infants were assigned to the placebo group. Questionnaire data were available for 317 children (71%) at a mean (SD) age of 5.8 (0.4) years (mean [SD] gestational age at birth, 29.3 [1.6] weeks; mean [SD] birth weight 1220 [340] grams; 128 [40%] female infants). At the age 5 years follow-up, the mean (SD) total difficulties score in the RHEpo group (8.41 [5.60] points) was similar to that of the placebo group (7.76 [4.81]) (*P* = .37). There were no statistically significant differences between the groups in any other outcome measures.

**Conclusions and Relevance:**

This secondary analysis of a randomized clinical trial showed no evidence for an effect of early high-dose RHEpo administration on behavioral outcomes or HRQoL in children born very preterm at early school age.

**Trial Registration:**

ClinicalTrials.gov Identifier: NCT00413946

## Key Points

**Question:**

Does erythropoietin improve behavior and health-related quality of life in children aged 5 years who were born very preterm?

**Findings:**

In this secondary analysis of a randomized clinical trial that included 448 infants born between 26 and 32 weeks’ gestational age randomized to high-dose erythropoietin or placebo within the first 48 postnatal hours, parent-rated behavior and health-related quality of life outcomes did not differ at age 5 years.

**Meaning:**

This randomized clinical trial found that prophylactic early high-dose erythropoietin did not affect behavior or health-related quality of life at age 5 years among children born very preterm.

## Teaser

This "prespecified secondary analysis of a randomized clinical trial investigates whether early high-dose recombinant human erythropoietin is associated with improved behavioral outcomes and health-related quality of life at age 5 years in children born very preterm.

## Introduction

During the last decades, the survival of preterm infants has steadily increased.^1^ While the number of preterm born children experiencing perinatal complications remains mainly unchanged,^2^ new strategies to improve neurologic outcomes in survivors are far from being established. The increased vulnerability of the immature brain^3^ results in an increased risk of long-term neurodevelopment impairments observed up to young adulthood involving mental, motor, and neurosensory functions,^4,5,6^ as well as psychiatric and behavioral, including socioemotional problems.^7,8^ These conditions are known to affect children’s quality of life up to young adulthood^9^ and their families’ environment.^10^

In light of the neuroprotective properties of erythropoietin (Epo) shown in preventing acute cerebral injury and promoting brain growth,^11^ the Swiss EPO Neuroprotection randomized clinical trial was initiated in 2005 to determine the neuroprotective effect of early high-dose recombinant human Epo (RHEpo) compared with placebo in infants born very preterm. While RHEpo was shown to be associated with improved white matter development in brain magnetic resonance imaging (MRI) examinations at term-equivalent age in a subgroup of the present study cohort,^12,13^ the primary outcome analysis showed no effect of RHEpo on the neurodevelopment at the corrected age of 2 years.^14^ These findings have been confirmed by others,^15^ and by a subsequent secondary outcome analysis of the cohort at age 5 years. The psychological adjustment after RHEpo of children born very preterm has not been reported yet, with the exception of a trial in which the early administration of erythropoiesis-stimulating agents (ie, RHEpo or darbepoetin) improved behavioral outcomes in a small cohort at age 3.5 to 4 years.^16,17^ In this study, we report the prespecified parent-reported behavioral outcome of the study infants at age 5 years and their HRQoL. The study hypothesis was that RHEpo results in favorable behavioral development and HRQoL compared with placebo.

## Methods

For this secondary analysis of a randomized clinical trial, data collection and evaluation were approved by the Ethical Committee of the Zurich University Children’s Hospital, by the Ethical Committee of the Canton Zurich, and by the Swiss Agency for Therapeutic Products (Swissmedic). Written informed consent was obtained from the parents of each participant. This study follows the Consolidated Standards of Reporting Trials (CONSORT) reporting guideline (Figure).

**Figure.**
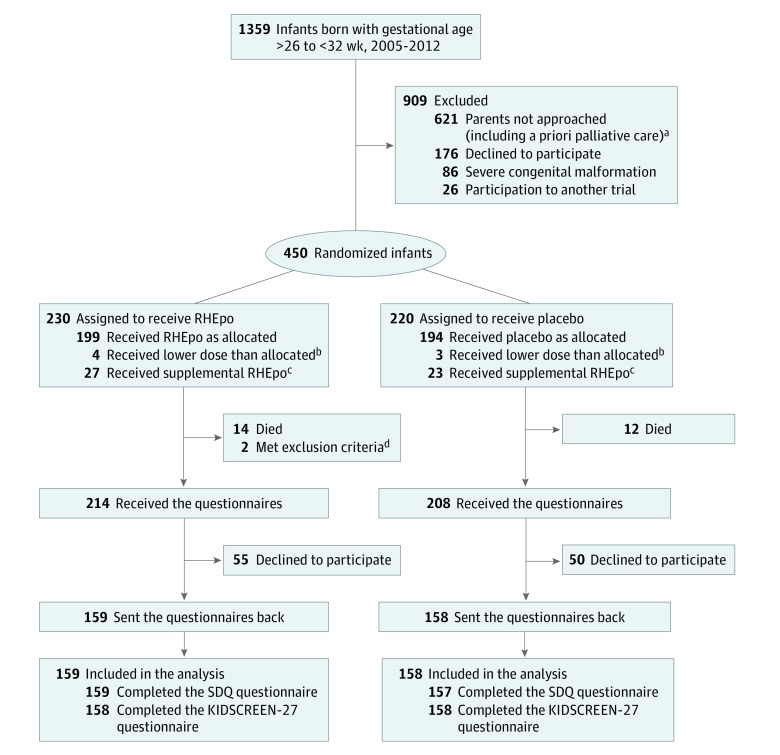
Participant Recruitment Flowchart KIDSCREEN-27 indicates the parent version of the KIDSCREEN-27 (KS-27) questionnaire used to evaluate health-related quality of life; SDQ, the parent version of the Strengths and Difficulties Questionnaire used to evaluate behavioral outcomes. ^a^Decision to provide primary nonintervention and palliative care after delivery was made antenatally with the agreement of the parents if several known prognostic factors were so unfavorable that the initiation of intensive care measures appeared to be inappropriate. ^b^Infants received lower than allocated dose after randomization, which occurred before 3 hours of life, exclusion criteria (5 infants), or nonadherence to inclusion criteria (attributable to errors in reporting of gestational age for 2 infants) were discovered for some infants; thus, they were excluded after randomization. ^c^Infants received supplemental recombinant human erythropoietin (RHEpo) to treat anemia of prematurity during the later neonatal course. ^d^Excluded after administration of the assigned treatment because of a uniparental disomy 16 and a dysmorphic syndrome that became evident after the third day of life (genetic investigations were still ongoing at the time of manuscript submission).

### Study Design and Population

In this phase III double-blind, placebo-controlled, multicenter randomized clinical trial, patients were enrolled within the first 3 hours after birth with 1:1 allocated to 2 intervention groups. Five Swiss perinatal centers (Aarau, Basel, Chur, Geneva, and Zurich) participated in the study. Enrollment occurred from 2005 to 2012 and included the participants at age 5 years. Infants born between 26 weeks 0 days’ and 31 weeks 6 days’ gestation were eligible for enrollment, while exclusion criteria were a genetically defined syndrome, severe congenital malformation adversely affecting life expectancy or neurodevelopment, severe intraventricular hemorrhage of greater than grade 2 before randomization, and a priori palliative care. The study protocol (Supplement 1),^18^ short-term safety outcomes of early high-dose RHEpo,^19^ and the primary and secondary outcomes at ages 2 and 5 years have been reported previously.^14,20^

### Neonatal and Sociodemographic Baseline Data

Neonatal baseline was defined as previously described.^14,19^ Socioeconomic status (SES) was estimated using a reliable and valid 12-point scale^21^ based on maternal education and paternal occupation, with 2 being the lowest score and 12, the highest.

### Intervention

The RHEpo intervention was r3 single doses of 3000 IU RHEpo (Roche) per kg body weight dissolved in 1 mL distilled water; the placebo control was an equivalent volume of sodium chloride 0.9% (placebo). Doses were administered intravenously within 3 hours, at 12 to 18 hours after birth, and at 36 to 42 hours after birth. The maximal dose was 1.5 mL (4500 IU; 37.5 μg) RHEpo for infants weighing 1.5 kg or more. The vials of the study medication were prepared in 1 study center (Zurich) according to a randomization list stratified per center then labeled, shipped, and stored in conformity with the Swiss Therapeutic Products Act. The study medication was randomly assigned to the patient number using a computer-based random-number generator. Multiples were randomized to the same treatment group. Parents, health care practitioners, and investigators were blinded to the treatment allocation.

### Procedure

Parents of the enrolled children were contacted via mail or by phone call when their offspring reached the age of 5 years. Parents were asked to complete 2 standardized questionnaires (German or French validated version) on the behavior and HRQoL (including growth parameters) of their child.

### Outcome Assessment

#### Behavioral Outcome

The parent version of the Strengths and Difficulties Questionnaire (SDQ) was used to evaluate the behavioral outcome of the children as a prespecified secondary outcome of the study. The SDQ is a standardized, open-access tool to screen for psychiatric and behavioral issues (ie, internalizing and externalizing disorders) in early school-aged children.^22^ Among the 5 subscales of the SDQ, 4 pertain to domains of child psychopathology: emotional symptoms, conduct problems, hyperactivity, and peer problems. An additional subscale relates to prosocial behavior. Each answer is scored using a Likert-type scale ranging from 0 (indicating not true) to 2 (indicating certainly true) with 5 of 25 items being reversely scored. The scores for each subscale are obtained by adding the corresponding 5 answers. Scale scores are to be computed only if at least 3 items per scale have been completed, imputing the missing items with the individual mean value of the answered items. The resulting subscales range from 0 to 10, with higher scores indicating more behavioral problems (or more pronounced prosocial behavior) and a higher risk for psychiatric pathology (or lower risk for prosocial problems). Excluding the prosocial scale, the remaining 4 scales on child psychopathology can be summed up in the total difficulties scale, ranging from 0 to 40, with higher score indicating more difficulties. Based on a large UK community sample,^23^ the scale scores are classified into 4 categories: close to average (corresponding to 80% of the population), slightly raised (10% of the population), high (5% of the population), and very high (5% of the population). The top 2 categories, high and very high, were combined into a single at-risk category.

### Health-Related Quality of Life

The parent version of the KIDSCREEN-27 (KS-27) questionnaire^24^ was used to evaluate the HRQoL of the children as an exploratory outcome of the study. The KS-27 is a standardized screening instrument for children and adolescents for the multidimensional assessment of HRQoL. It is composed of 27 items regarding 5 dimensions of HRQoL: physical well-being, psychological well-being, autonomy and relationship with parents, social support, and school environment. Each item is rated on a 5-point scale. Since the questionnaire standardization exists only for the age range 8 to 18 years, coding the scores of each domain into *T*-values was not possible. Therefore, after recoding the 4 negatively formulated items, raw scores for all items in each dimension were summed and transformed into a 0 to 100 continuum, with higher values indicating better HRQoL, as previously reported for this questionnaire.^25^ Missing data were imputed per mean only if 1 item per scale was missing, according to the KIDSCREEN Manual,^26^ otherwise the entire subscale was set as missing.

### Statistical Analysis

A minimal sample size of 176 infants per group was calculated based on the primary outcome at age 2 years, and 20% were added targeting at least 211 infants per group to compensate for dropouts, as previously reported.^14^ Baseline characteristics between study groups were compared through generalized estimating equations to control for statistical dependencies of same-birth siblings. The internal consistency of questionnaires was calculated by means of Cronbach α. Item-level missing data were imputed according to the respective questionnaire manuals.

The investigation was an unadjusted comparison between groups, including participants as originally randomized. The model assumption was that the observations in both groups come from a normal distribution. This assumption was not violated. The unadjusted treatment effect was determined using generalized estimating equations to account for the cluster structure of outcomes with same-birth siblings. Because we used an exchangeable working correlation structure for continuous outcomes, the estimated mean differences differ slightly from the mean group’s differences. For binary or ordinal outcomes, we used an independence working correlation structure. A comparison between groups of participants including only children who completed the allocated treatment was performed as a sensitivity analysis with the same methods. Results concerning the outcomes were reported as mean differences or odds ratios with 95% CIs. Statistical analyses were performed using R version 4.1.1 and the R package *geepack*^27^ version 1.3.4 (R Project for Statistical Computing). The significance threshold was defined as 2-sided *P* < .05. Data were analyzed from January 6 to December 31, 2021.

## Results

### Study Population

Among 1359 eligible infants born during the study period, 450 infants (mean [SD] gestational age at birth, 29.3 [1.6] weeks; mean [SD] birth weight 1220 [340] grams; 128 [40%] female infants) were randomized, with 228 allocated to the RHEpo group and 220 allocated to the placebo group. Two randomized infants (allocated to the RHEpo group) were retrospectively excluded because of a later diagnosis of a congenital syndrome. For this analysis, secondary outcome data were available for 159 children (70%) in the RHEpo group and 158 children (72%) in the placebo group, at a mean (SD) age of 5.8 (0.4) years. The neonatal and sociodemographic baseline characteristics did not differ between the groups (Table 1). Among all randomized infants, baseline characteristics of participants and nonparticipants in this follow-up study were comparable, with the exception of a lower rate of major brain lesions and a higher umbilical artery pH in participants than in nonparticipants (Table 1). In addition, families of participants had a higher socioeconomic status than those of nonparticipants. Of 317 children included in the analyses, 36 infants were not treated as intended in the study plan. The rate of infants with lower dose of RHEpo (3 infants) or placebo (3 infants) than allocated, and the rate of infants with supplemental RHEpo to treat anemia of prematurity during the neonatal course (17 infants in the RHEpo group and 13 infants in the placebo group) were similar between groups.

**Table 1.** Perinatal and Sociodemographic Characteristics of Study Population and Their Families

Characteristics	Participants, No. (%)		
	RHEpo (n = 159)	Placebo (n = 158)	Nonparticipants (n = 131)
Gestational age, mean (SD), wk	29.1 (1.6)	29.4 (1.6)	29.0 (1.7)
Sex			
Female	65 (41)	63 (40)	57 (44)
Male	94 (59)	95 (60)	74 (56)
Singletons	104 (65)	106 (67)	71 (54)
Birth weight, g			
Mean (SD)	1200 (328)	1240 (352)	1186 (349)
*z*-score, mean (SD)	–0.11 (0.75)	–0.08 (0.86)	–0.11 (0.84)
Birth head circumference, cm			
Mean (SD)	27.0 (2.1)^a^	27.1 (2.3)^a^	26.5 (2.2)
z-score, mean (SD)	–0.08 (0.68)^a^	–0.14 (.74)^a^	–0.23 (0.66)
Antenatal steroids	152 (96)^b^	150 (95)^a^	121 (92)^c^
Chorioamnionitis	45 (28)^b^	43 (27)^a^	33 (25)^c^
Apgar at 5 min, median (IQR)	8 (7 to 9)	8 (6 to 9)^a^	8 (6 to 9)^c^
Umbilical artery pH, mean (SD)	7.32 (0.08)^d^	7.32 (0.08)^e^	7.30 (0.1)^f^
Mechanical ventilation, median (IQR)	0 (0 to 3)	0 (0 to 3)^a^	1 (0 to 4)^c^
Hypoglycemia	65 (41)^b^	73 (46)^g^	54 (41)^c^
Bronchopulmonary dysplasia	56 (35)	51 (32)	36 (28)
Necrotizing enterocolitis	3 (2)	5 (3)	8 (6)
Sepsis	22 (14)	22 (14)	15 (12)
Major brain lesions^h^	4 (3)^b^	8 (5)^a^	17 (13)^c^
Retinopathy of prematurity >2° grade	1 (1)	3 (2)	3 (2)
Patent ductus arteriosus	42 (26)	53 (34)	32 (24)
Mother’s age at delivery, mean (SD), y	32.3 (4.7)	33.0 (5.4)^a^	32.1 (6.2)^c^
Socioeconomic status score, median (IQR)	6 (4to 7)^i^	5 (4 to 6)^i^	6 (4 to 8)^j^
Child’s age at follow-up, mean (SD), y	5.8 (0.4)	5.8 (0.5)	NA

Abbreviation: NA, not applicable.

^a^ Includes 157 participants.

^b^ Includes 158 participants.

^c^ Includes 12 participants.

^d^ Includes 146 participants.

^e^ Includes 142 participants.

^f^ Includes 120 participants.

^g^ Includes 156 participants.

^h^ Major brain lesion, ie, intraventricular hemorrhage grade 2 or greater and/or cystic periventricular leukomalacia.

^i^ Includes 153 participants.

^j^ Includes 116 participants.

### Behavioral Outcome

At the age 5 years follow-up, the mean (SD) total difficulties score in the RHEpo group (8.41 [5.60] points) was similar to that of the placebo group (7.76 [4.81]; *P* = .37). Cronbach α was 0.77 for the hyperactivity subscale and 0.80 for the total difficulties score. For the other subscales Cronbach α ranged from 0.53 to 0.68. Among all SDQ questionnaires, 0.3% of unanswered items were found and imputed, accordingly. The scores of the 5 subscales and the total difficulties scale of the SDQ, and the respective rates of children considered outside the reference ranges were similar in both groups (Table 2).

**Table 2.** Comparison of Parent-Reported Behavioral Problems According to the SDQ Between the Study Groups at Age 5 Years

SDQ Scale	RHEpo (n = 159)	Placebo (n = 157)	Mean difference (95% CI)	OR (95% CI)	*P* value	Cronbach α
Emotional problems^a^						
Overall, mean (SD)	2.04 (2.04)	1.69 (1.70)	0.36 (<0.00 to 0.71)	NA	.10	0.65
With at-risk score, No. (%)^b^	18 (11)	12 (8)	NA	1.54 (0.80 to 2.97)	.28	NA
Conduct problems^a^						
Overall, mean (SD)	1.64 (1.50)	1.76 (1.51)	–0.17 (–0.47 to 0.13)	NA	.35	0.53
With at-risk score, No. (%)^b^	20 (13)	23 (15)	NA	0.84 (0.48 to 1.46)	.60	NA
Hyperactivity^a^						
Overall, mean (SD)	3.28 (2.48)	3.18 (2.28)^c^	0.09 (–0.37 to 0.55)	NA	.75	0.77
With at-risk score, No. (%)^b^	17 (11)	8 (5)^c^	NA	1.32 (0.68 to 2.53)	.49	NA
Peer problems^a^						
Overall, mean (SD)	1.44 (1.87)^c^	1.14 (1.47)	0.30 (–0.02 to 0.62)	NA	.13	0.66
With at-risk score, No. (%)^b^	19 (12)^d^	13 (8)	NA	1.51 (0.81 to 2.83)	.27	NA
Prosocial behavior^e^						
Overall, mean (SD)	8.12 (1.72)	7.97 (1.79)	0.21 (–0.12 to 0.55)	NA	.29	0.68
With at-risk score, No. (%)^b^	5 (3)^d^	6 (4)	NA	0.82 (0.30 to 2.25)	.75	NA
Total difficulties^f^						
Overall, mean (SD)	8.41 (5.60)^d^	7.76 (4.81)^c^	0.55 (–0.45 to 1.55)	NA	.37	0.80
With at-risk score, No. (%)^b^	13 (8)^d^	8 (5)	NA	1.67 (0.74 to 3.78)	.30	NA

Abbreviations: NA, not applicable; RHEpo, recombinant human erythropoietin; SDQ, Strengths and Difficulties Questionnaire.

^a^ Score range, 0-10; higher scores indicate more behavioral problems.

^b^ At-risk score corresponds to the top 10% of scores outside of reference range of each scale, based on a large UK community sample.^23^

^c^ Includes 156 participants.

^d^ Includes 158 participants.

^e^ Score range, 0 to 10; lower scores indicate more behavioral problems.

^f^ Score range, 0 to 40; higher scores indicate more behavioral problems.

### Health-Related Quality of Life

There were no significant differences between groups in HRQoL. Cronbach α values ranged from 0.74 to 0.81 in all dimensions of KS-27, except for the autonomy and parent relation (mean difference, 2.34; 95% CI, –10.24 to 14.91; α = 0.67). Among all questionnaire items, 2.8% were unanswered. In particular, 17.1% of the autonomy and parent relation scale was set as missing, with 2 answers (ie, “Has your child had enough money to do the same things as his/her friends?” and “Has your child felt that he/she had enough money for his/her expenses?”) missing in approximately 20% of participants. Scores of the 5 KS-27 dimensions were similar between groups (Table 3). Sensitivity analyses including only study participants who completed the allocated treatment (per protocol analysis) revealed similar findings with respect to the SDQ and KS-27 measures (eTable 1 and eTable 2 in Supplement 2).

**Table 3.** Comparison of Parent-Reported Health-Related Quality of Life According to the KIDSCREEN-27 Between Groups at Age 5 Years

KIDSCREEN-27 Questionnaire Dimensions^a^	Mean (SD) score		Mean difference (95% CI)	*P* value	Cronbach α
	RHEpo (n = 158)	Placebo (n = 158)			
Physical well-being	74.71 (12.54)^b^	76.60 (13.28)	–1.75 (–4.22 to 0.72)	.24	0.80
Psychological well-being	85.46 (9.29)^b^	86.49 (9.15)^b^	–0.11 (–1.83 to 1.60)	.91	0.74
Autonomy and parent relation	82.77 (9.81)^c^	82.47 (12.05)^d^	2.34 (–10.24 to 14.91)	.76	0.67
Social support and peers	74.05 (15.86)^e^	74.96 (15.43)^f^	–0.73 (–3.60 to 2.14)	.68	0.82
School environment	86.00 (12.62)^e^	86.10 (13.10)^g^	0.76 (–1.83 to 3.35)	.63	0.81

Abbreviation: RHEpo, recombinant human erythropoietin.

^a^ Raw scores for all items in each dimension were summed and transformed in a 0 to 100 continuum, with higher values indicating better quality of life.

^b^ Includes 157 participants.

^c^ Includes 132 participants.

^d^ Includes 130 participants.

^e^ Includes 154 participants.

^f^ Includes 153 participants.

^g^ Includes 147 participants.

## Discussion

In this prespecified secondary analysis of a multicenter, double-blinded randomized clinical trial enrolling very preterm infants born between 26 weeks 0 days’ and 31 weeks 6 days’ gestation treated either with prophylactic early high-dose RHEpo or saline during the first 2 days after birth, we observed no relationship between the treatment and behavioral outcomes and HRQoL at age 5 years. While these results do not support the study hypothesis, they are in line with those of the previous primary and secondary outcome analyses of the Swiss EPO Neuroprotection Trial,^14,20^ which also found RHEpo had no impact on the neurodevelopment up to the age of 5 years, and with those of another large trial.^15^

Previous literature reported neuroprotective properties of Epo in both preclinical and clinical settings.^11^ However, any comparison with other studies on prophylactic early RHEpo for neuroprotection in the preterm newborn needs to take into consideration the relevant differences in the RHEpo dosage between the Swiss EPO Neuroprotection Trial and other trials, in which 1000 U/kg RHEpo or less was repeatedly administered over 2 weeks or longer after birth.^15,28,29,30^ Additionally, all published trials to our knowledge have been designed for studying the effect of RHEpo on cognitive performance, and very few of them reported outcomes following early infancy. A 2021 meta-analysis by Fischer et al^31^ on the association of prophylactic RHEpo on neurodevelopment in very preterm infants, which included 1796 infants from 6 randomized clinical trials (including the Swiss EPO Neuroprotection Trial), found a significant reduction of patients with poor cognitive scores at age 18 to 24 months with a number needed to treat of 17. However, Fischer et al^31^ reported a substantial study heterogeneity, attributable to a single study featuring a high risk of bias, which questioned the objective effect of RHEPO treatment. To our knowledge, only 1 study has reported behavioral outcomes at age 4 years in a small cohort of very low–birth weight babies, in which treatment with erythropoiesis stimulating agents (RHEpo or darbepoetin) resulted in less behavioral symptoms and externalizing problems as assessed by the Behavioral Assessment of Children Scale–Second Edition.^16^ Contrary to our findings, a randomized clinical trial by Wu et al^32^ found that high-dose RHEpo led to increases in externalizing problems in children aged 2 years who had experienced moderate or severe neonatal hypoxic ischemic encephalopathy.^32^

The lack of an effect of RHEpo on the parent-reported HRQoL of the study children refutes the study hypothesis. HRQoL is a key metric that quantifies how a clinical condition or treatment affects the physical, socioemotional, and psychological well-being of a patient. In addition to long-term functional milestones measurements, multidimensional HRQoL measurements become increasingly important for newborns at high-risk of adverse outcomes and their relatives, especially following trials of medicinal products.^33^ Given the previously reported association between RHEpo and favorable white matter development at term equivalent in a subset of the Swiss EPO Neuroprotection Trial,^12^ and previous literature,^11^ it was expected that a neurodevelopmental advantage would translate into a benefit on HRQoL in childhood. However, since no significant difference was observed between the RHEpo group and the placebo group in the neonatal morbidities^19^ or in the primary and secondary neurodevelopmental outcomes at ages 2 and 5 years,^14,20^ the present finding is not surprising.

To explain the absence of a link between RHEpo and the measured outcomes in this study, we formulate 3 hypotheses. First, given the differences in the dosage of RHEpo between the Swiss EPO Neuroprotection Trial and the other trials studying the neuroprotective effect of RHEpo in very preterm infants, we assume that prophylactic short-term high-dose RHEpo administered early after birth to all infants born preterm does not affect neurodevelopment. While there is no conclusive data on the optimal RHEpo administration for neuroprotection in newborn infants, animal models show that high-dose RHEpo could be needed to cross the blood brain barrier^34,35^ and that the duration of therapy seems to be positively associated with better neurologic outcomes.^35,36^ Second, since the endogenous regulation of the Epo receptor expression in neurons, astrocytes, and microglia increases in response to hypoxia,^37^ it may be that RHEpo is more effective as neuroreparative agent when administered after an acute brain damage.^38^ Therefore, the target population of this study may not represent the population of infants with the highest risk for brain lesions and neurodevelopmental impairment who would have benefited from a putative neuroprotective effect of RHEpo. The results of the ongoing EpoRepair Trial^39^ will show whether RHEpo plays a neuroprotective role in very preterm infants with intraventricular hemorrhage at age 5 years. Finally, it is possible that RHEpo does not impact the neurodevelopment of children born very preterm. Contrary to this hypothesis, early high-dose RHEpo administration was positively associated with an improvement in white matter development in a subgroup of the present cohort, as assessed by means of cranial MRI at term equivalent age,^12,13^ which has been also reported by others.^40^ It remains unclear to which extent the observed benefit of RHEpo on structural brain development is related to measurable functional outcomes in very preterm infants. An ongoing follow-up study of the Swiss EPO Neuroprotection cohort will test whether the previously observed improvement of white matter development after early RHEpo exposition is confirmed by brain imaging and reflected in better executive function abilities at age 7 to 12 years.^41^ The strengths of this study include its double-blinded, placebo-controlled randomized design, the size of its sample and the use of a standardized multidomain measurement of behavioral functioning at early school age.

### Limitations

This study has some limitations. First, an attrition bias may have arisen due to the loss of 29% of the originally randomized infants. This selection resulted in a lower rate of major brain lesions and a higher socioeconomic status in participants than in nonparticipants, and further reduced the already low-risk profile of the studied population. Second, 11% of children who participated in the follow-up had received the supplemental or insufficient administration of RHEpo than allocated. Since the intention-to-treat and the per-protocol analyses revealed similar results, and since the rates of protocol violation events were similar between groups, the related bias may be assumed to be low. The third limitation is the use of parent-reported measures of behavior and HRQoL only. Indeed, although standard measurement methods were used for both outcomes, the lack of self-reported results by the participants and the lack of clinical data on their health status could have caused informational bias. However, the divergences between children’s and parental reports on child health-related assessments, which are known in the literature, rather suggest different perspectives and not necessarily inaccuracies or biases.^42^ Additionally, some subscales of the behavioral and HRQoL measures showed low reliabilities that may affect the interpretation of the results. While moderate reliabilities for the SDQ subscales have been reported before across studies,^43^ the low reliability and the missing data for the HRQoL subscale autonomy and parent relation likely reflects an age-inappropriateness for some of the items.^44^ While results for this subscale should be interpreted with caution, the overall pattern and interpretation of the results do not change if it is excluded.

## Conclusions

In this secondary analysis of a randomized clinical trial, no effects of prophylactic early high-dose RHEpo administered over the first 2 days of life on parent-reported behavioral outcomes or HRQoL were observed in very-preterm–born children at age 5 years. The findings of an ongoing follow-up study that evaluates this cohort up to the age of 12 years will show whether relevant clinical neurodevelopmental outcomes after RHEpo would become evident later in life.

## Notes

Supplement.
Trial Protocol and Statistical Analysis Plan

Supplement 2. **eTable 1. **Comparison of Parent-Reported Behavioral Problems According to the SDQ Between Groups at Age 5 Years Restricted to Children Who Completed the Allocated Treatment **eTable 2.** Comparison of Parent-Reported HRQoL According to the KIDSCREEN-27 Between Groups at Age 5 Years Restricted to Children Who Completed the Allocated Treatment

Supplement 3.
The Swiss EPO Neuroprotection Trial Group

Supplement 4.
Data Sharing Statement
