# Supplementary material for: Effect of Early High-Dose Recombinant Human Erythropoietin on Behavior and Quality of Life in Children Aged 5 Years Born Very Preterm: Secondary Analysis of a Randomized Clinical Trial
Source: JAMA Netw Open. 2022 Dec 7;5(12):e2245499. doi: 10.1001/jamanetworkopen.2022.45499 (PMC9856490; doi:10.1001/jamanetworkopen.2022.45499)
Supplement: Supplement. — Trial Protocol and Statistical Analysis Plan [file jamanetwopen-e2245499-s001.pdf]

Study Protocol (StV- 36/04)

Does Erythropoietin improve outcome in very preterm infants?

A Randomised, Double-blind, Placebo-controlled, Prospective, Multicenter Clinical Study

**Short title of clinical study**	Epo
**Version**	17
**Date of Protocol Amendments**	17 September 2008<br>A1 vom 15.10.2005<br>A2 vom 28.2.2007<br>A3 vom 30.6.2008<br>A4 vom 17.9.2008
**Sponsor of the Study**	Prof. Dr. med. Hans Ulrich Bucher, Zürich
**Principal investigators**	1. PD Dr. med. Riccardo Pfister, Genève<br>2. PD Dr. med. Mathias Nelle, Berne<br>3. Prof. Dr. med. C. Bührer, Basel<br>4. PD Dr. med. Jean-Claude Fauchère, Zürich<br>5. Fr. Dr. med. Sylviane Pasquier<br>6. Fr. Dr. med. Brigitte Scharrer
**Investigational drug**	Erythropoietin

Table of Contents

1. General Information **4**
1.1 Responsible Staff *4*
1.2 Scientific Advisors *4*
1.3 Ethical Committee Responsible for the Primary Investigator *5*
1.4 Participating study centres and local investigators *5*
1.5 Protocol Synopsis *6*
1.6 Time table *7*
1.7 Diagram of study procedures *8*
2. Introduction **8**
2. Introduction **9**
2.1 Summary *9*
2.2 Background *10*
3 Study Objectives **12**
3.1 Hypothesis *12*
3.2 Primary Objective *12*
3.3 Definition of the Primary Objective *12*
3.4 Secondary Objectives *12*
4. Study design **12**
4.1 Design *12*
4.2 Randomisation *12*
4.3 Study duration *12*
5. Study Population **13**
5.1 Recruitment *13*
5.2 Inclusion criteria *13*
5.3 Exclusion criteria *13*
6. Study medication **13**
6.1 Investigational drug *13*
6.2 Blinding, Packaging, Storage Instructions, Labelling, Shelf Life *14*
6.3 Undesirable effects *14*
6.4 Description of Verum Treatment *14*
6.5 Description of Placebo Treatment *14*
6.6 Allowed/Disallowed Concomitant Medication *15*
6.7 Reference Sample *15*
7. Study Procedures and Examination Methods **15**
7.1 Inclusion in the Study, Beginning of Study *15*
7.2 Study-related Procedures *15*
7.3 Study-related Tests and Examinations *16*
7.4 Magnetic resonance imaging *16*
7.5 Individual Withdrawal Criteria *19*
7.6 Individual End of the Study *19*
7.7 Patient's consent, information and data protection declaration *19*
7.8 Follow up Study *19*
7.9 Randomisation and Deblinding *20*
8. Methods for Assessing Efficacy and Safety **20**
8.1 Appraisal of the Risk-to-Benefit Ratio *20*
8.2 Methods for Assessing Efficacy *20*
8.3 Adverse Events (AE), Definition *21*
8.4 Serious Adverse Events (SAE), Definition *21*
8.5 SAE, AE: Evaluation, Assessment, Documentation *21*

8.6 Premature Termination of the Clinical Trial *21*
9. Information on Statistics, Evaluation 22
9.1 Number of Cases, Statistical Considerations *22*
9.2 Biometric Planning and Data Evaluation *22*
9.3 Interim Analysis *23*
10. Data Collection Forms, Data Management 23
10.1 Data Collection Forms *23*
10.2 How will individual findings be collected and documented? *23*
10.3 Patient Identification List *23*
10.4 Filing of Study Records *24*
11. Ethical Issues, Data Protection, Quality Assurance, Insurance 24
11.1 ICH/GCP Guidelines, the Helsinki Declaration, Legal Provisions *24*
11.2 Assessment of the Protocol by the Responsible Ethics Committee *24*
11.3 Handling of Additions/Changes to the Protocol *24*
11.4 Information for Parents *24*
11.5 Data Protection Statement *24*
11.6 Monitoring, Inspections *25*
11.7 Insurance *25*
12. General Provisions, Agreements, Organisational Procedures 25
12.1 Reports to Authorities *25*
12.2 Final Report *25*
12.3 Publications *25*
13. Literature 26

1. General Information

1.1 Responsible Staff

Primary Investigator: Prof. H.U. Bucher, Klinik für Neonatologie, UniversitätsSpital, 8091 Zürich
Phone: 044 255 53 40, Fax: 044 255 44 42, E-mail: buh@usz.ch

Finances: Frau Anita Faure, Klinik für Neonatologie, UniversitätsSpital, 8091 Zürich
Phone: 044/ 255 53 40, Fax. 044/ 255 44 42 ,
Email: anita.faure@usz.ch

Study Coordinator: Frau B. Koller, Klinik für Neonatologie, UniversitätsSpital, 8091 Zürich
Phone: 044/ 255 93 27, Fax: 044/ 255 44 42
Email: brigittemaria.koller@usz.ch

Data Management : Lic. phil. Marc Adams, Klinik für Neonatologie, UniversitätsSpital Zürich, 8091 Zürich
Phone: 044 255 53 40, E-mail: Marc.Adams@usz.ch

Pharmacy: Dr. D. Fetz Kantonsapotheke Zürich, Spöndlistrasse 9, 8006 Zürich
Phone : 044 255 32 19, Fax : 044 255 45 46
E-Mail: Daniel.fetz@ksa.ch

Consulting Biometrician: Dr. phil. A. Tschopp, Abteilung für Biostatistik, ISPM, Universität Zürich, Sumatrastrasse 30, 8006 Zürich
Phone: 044 634 46 42, Email: Alois.tschopp@ifspm.unizh.ch

Safety Monitoring Board : Fr. Dr. M. Mönkhoff, Abteilung Neonatologie, Regionalspital Zollikerberg
Phone: 044 397 22 25 Fax: 044 397 25 77
Email: Marion.Moenkhoff@spitalzollikerberg.ch

Analysis of MRI-Data : Prof. P. S. Hüppi, Unité de Développement, Hôpital des Enfants, 6 rue Willy Donzé, 1211 Genève 14
Phone 022 372 43 52, Fax 022 372 43 15
Email : petra.huppi@hcuge.ch

1.2 Scientific Advisors

Prof. Christian Bauer, Emeritus, ehemals Institut für Physiologie, Universität Zürich

Prof. Christof Dame. Juniorprofessor, Klinik für Neonatologie, Charité Berlin

Max Gassmann. Full Professor of Veterinary Physiology and Director of the Institute of Veterinary Physiology, University of Zürich

Christian Grimm. PD Dr. phil. Laboratory for Retinal Cell Biology
Eye Clinic University of Zurich Hospital

Prof. Hugo Marti, Universität Heidelberg, Institut für Physiologie und Pathophysiologie, Im Neuenheimer Feld 326, D-69120 Heidelberg

1.3 Ethical Committee Responsible for the Primary Investigator

1) Ethik-Kommission am Kinderspital Zürich (SPUK)

   Prof. Dr. med. E. Schönle (Chair)

   Universitäts-Kinderklinik

   Steinwisstrasse 75, 8032 Zürich

   Tel. 044 266 75 97 Fax: 044 266 79 83

   E-mail: eugen.schoenle@kispi.uzh.ch

2) Kantonale Ethikkomission (KEK)

   Prof. Dr. med. R. Maurer (Chair), Niklaus Herzog, lic. iur. et theol.

   Obstgartenstrasse 21, 8090 Zürich

   Tel.: 043 259 5211 Fax: 043 259 5163

   E-mail: niklaus.herzog@gd.zh.ch

Participating study centres and local investigators

Genève:	PD Dr. med. Riccardo Pfister
Bern:	PD Dr. med. Mathias Nelle
Basel:	Prof. Dr. med. Christoph Bührer
Aarau:	Dr. med. Sylviane Pasquier
Zürich:	PD med. Dr. Jean-Claude Fauchère
Chur:	Dr. med. Brigitte Scharrer

Protocol Synopsis

Study code	EPO
Protocol title	Does erythropoietin improve outcome in very preterm infants?
Study design	Multicentre, placebo-controlled, prospective, randomised, double-blind (verum:placebo = 1:1) Study
Planned sample size	Total number 420 preterm infants
Calculation of case numbers	176 cases per group (2-sided alpha 0.05, power 0.8)
Study duration	Scheduled starting date: 01/2006<br>Prospective total duration of study 24 months + 24 mths follow-up<br>Study duration (clinical part) / patient max. 16 weeks until 40.th week of gestation
Question	Does erythropoietin reduce brain, eye, lung and gut damage and ameliorate the neurodevelopmental outcome in preterm infants?
Primary Objective	Mental development at 24 months (Bayley II)
Secondary Objectives	1. Incidence of intracranial haemorrhage<br>2. white matter disease (periventricular leucomalacia)<br>3. septicaemia<br>4. necrotising enterocolitis<br>5. persistent ductus arteriosus (PDA)<br>6. bronchopulmonary dysplasia<br>7. retinopathy<br>8. Length of stay in hospital<br>9. Cerebral volume at 40 postmenstrual weeks (MRI)<br>10. Weight, length and head circumference at 40 postmenstrual weeks and 24 months after term<br>11. Incidence of visual, hearing and motor impairment at 24 months
Inclusion Criteria	1. Infants born between 26 $^{0}/_{7}$ and 31 $^{6}/_{7}$ gestational weeks<br>2. Postnatal age less than 3 hours<br>3. Informed parental consent (preferably obtained before birth)
Exclusion Criteria	1. Genetically defined syndrome<br>2. Severe congenital malformation adversely affecting life expectancy<br>3. Severe congenital malformation adversely affecting neurodevelopment<br>4. A priory palliative care<br>5. Intracranial haemorrhage grade 2 or more
Investigational drug	Human erythropoietin, intravenous applicationDosage: 3000 U/kg of body weight, Administered three times, at 3 hours, 12 to 18 hours and 36 to 42 hours after birth.
Comparison therapy	NaCl 0.9%, same administration route and volume as investigational drug

1.6 Time table

Point(s) of time / Action	Before start of study	In Hospital				Follow-up	
		Day 1 -3	Day 7 to 10	Every two gestational weeks	Discharge home	Gestation week 40	24 months corrected for prematurity
**Inclusion criteria**	●						
**Exclusion criteria**	●						
Demographic data	●						
**Vital signs**							
Continuous monitoring[1]	●	●	●	o			
**Laboratory**							
Blood count[2]	●	o	●	o			
Clinical chemistry[3]	o	o	o	o			
Placenta Histology		o					
**Ventilation/CPAP**							
Respiratory support	●	●	●	●			
Ventilation parameters	●	●	●	●			
**Diagnostics**							
Epo concentration[4]	●						
Cerebral ultrasound	●	●	●	●	●		
Chest X-ray		●	o	o	o		
Brain MRI						o	
Serum, csf cultures	o	o	o	o			
**Therapy**							
Antibiotics	●	●	●	●			
Catecholamines	●	●	●	●			
other medication[5]	●	●	●	●			
**Study medication**							
administration of investigat. drug		●●●					
**Adverse events**		●	●	●	●		
**Serious adverse events**		●	●	●	●		
Confirmation of completeness					●		●

- ● compulsory (for medication, adverse events etc indicate „no“ if none), o optional

1. Continuous monitoring: heart rate, pulse oximetry, (optional: transcutaneous pO2 and pCO2, arterial blood pressure, cerebral oxygenation with Near infrared Spectroscopy [NIRS] optional)
2. Blood counts: haematocrit; leucocytes, thrombocytes and differential blood count only when medically indicated
3. Clinical chemistry: Na, K; creatinine, urea (only when medically indicated))
4. Epo concentration in umbilical cord serum
5. Other medication: aminophylline, caffeine, insuline, indomethacine or ibuprofen, steroids etc.

1.7 Diagram of study procedures

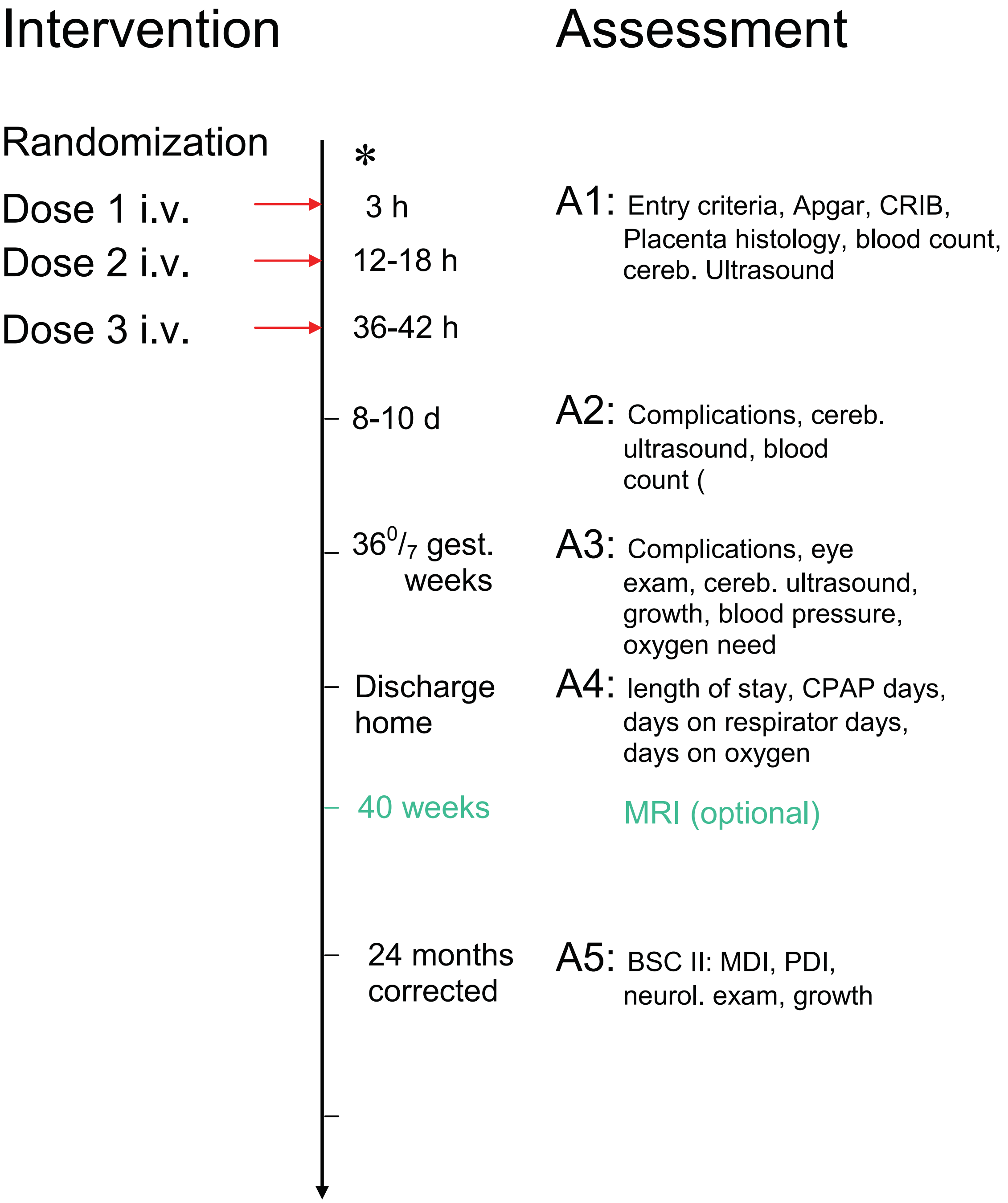

2. Introduction

Summary

Hypothesis

Early administration of human erythropoietin (EPO) in very preterm infants reduces perinatal injury to the brain (retina), lung and gut and improves neurodevelopmental outcome at 24 months corrected age.

Primary objective

To determine whether cerebral outcome is improved if infants born between 26 $^{0}/_{7}$ and 31 $^{6}/_{7}$ gestational weeks at birth receive erythropoietin in high dose in the first three days after birth.

Secondary objectives

To determine whether early administration of EPO alters the incidence of complications typically associated with preterm birth, i.e. mortality, septicaemia, necrotising enterocolitis, bronchopulmonary dysplasia (oxygen dependency at 36 weeks postmenstrual age), retinopathy, intracranial haemorrhage, white matter disease (periventricular leucomalacia), growth failure, cerebral palsy and handicap at 5 years.

Rationale

EPO has been shown to be protective against hypoxic-ischaemic and inflammatory injuries in a broad range of tissues and organs besides promoting red cell formation. It has been shown to have neuroprotective and neurotrophic activity in animals after acute brain damage as well as in adult stroke patients. Several mechanisms explaining this activity have been recognized: EPO inhibits glutamate release in the brain, modulates intracellular calcium metabolism, induces the generation of anti-apoptotic factors, reduces inflammation, decreases nitric oxide–mediated injury, and has direct antioxidant effects.

Very preterm infants have significant delay in mental and physical development assessed at 24 months corrected age. The most critical period are the first days after preterm birth where the oxygenation of the brain may be impaired by respiratory, circulatory and nutritional insufficiency. Although there are probably several mechanisms involved in permanent brain damage, it is most likely that EPO with its multiple action may reduce this damage.

EPO has been studied in several trials in preterm infants to prevent anaemia and is now widely used for this indication.

Study design

Randomized, double-masked, placebo-controlled multi-center clinical trial.

Research plan

420 infants will be randomized during the first three hours of life to receive EPO (3000 U/kg body weight) or placebo intravenously at 3, 12-18 and 36-42 hours after birth. Standardized evaluation including cerebral sonography at day 1 and 7-10 and at 36 $^{0}/_{7}$ gestational weeks (or at discharge home if discharged before) will determine the presence or absence of complications. Cerebral volume and white matter volume will be assessed at 40 postmenstrual weeks with MRI (only if available).

Experienced examiners will assess developmental function at 24 months corrected age using the reliable and validly revised Bayley Scales II of Infant Development and determine the presence or absence of impairment of motor function (cerebral palsy) and neurosensory function (blindness or deafness).

At 5 years corrected age KaufmannABC II (a standardized neuropsychological test), neurological examination (cerebral palsy), visual and hearing examination, growth assessment and a questionnaire on child behavior will be used.

Clinical significance

At least 1 of every 100 liveborn infants is born very preterm. 90% of these infants survive but >50% have a delay in mental and physical development assessed at 24 months corrected age. More subtle problems affecting cognition, vision and hearing are common at the age of five years and have an impact on school performance and quality of life of the infants and their families. The aim of this trial is to examine whether a short, easily applicable and well tolerated pharmacological intervention can improve neurodevelopmental outcome.

2.2 Background

At least 1 of every 100 live born infants are very preterm. 90% of these infants survive, but the incidence of complications such as intracranial haemorrhage, periventricular leukomalacia, necrotizing enterocolitis, bronchopulmonary dysplasia, persistent ductus arteriosus and septicaemia is high. A considerable proportion of the afflicted infants develop cerebral palsy, delayed mental development and visual impairment when assessed at 24 months corrected age. More subtle problems affecting cognition, vision and hearing are common at the age of five years and have an impact on school performance and quality of life. The aim of this study is to examine, whether a short, easily applicable and well tolerated pharmacological intervention can improve outcome of important complications of prematurity.

2.2.1 Cell Culture and Animal Experiments

EPO is a haematopoietic growth factor and cytokine which stimulates not only erythropoiesis but also has a critical role in the development, maintenance, protection and repair of the nervous system as well of other organs. A wide variety of experimental studies have shown that EPO and its receptor are expressed in the central nervous system and EPO exerts remarkable neuroprotection in cell cultures and animal models of nervous system disorders. The same is true for protective effects in other organs. The current knowledge on the mechanisms by which EPO produces neuroprotection and the signal transduction system regulated by EPO in the nervous system are summarized in two recent reviews[5,6].

The most relevant facts for the proposed study are briefly summarized: Studies in rats and primates showed that EPO in high dose crosses the blood brain barrier and has neuroprotective and neurotropic activity[7,8]. Several mechanisms explaining this activity have been recognized: EPO inhibits glutamate release, modulates intracellular calcium metabolism, induces the generation of neuronal anti-apoptotic factors, reduces inflammation, decreases nitric oxide-mediated injury, and has direct antioxidant effects[9]. The most often used animal model are rats in whom several research groups could demonstrate a significant decrease in infarct volume after a hypoxic-ischaemic insult and an improvement in task resolving and memory after a single dose of EPO[10,5,11,12,13,14,15,16].

EPO receptors have been demonstrated not only in the bone marrow, spleen, liver and brain but in many other organs such as the small bowel[17]. EPO has been shown to protect against necrotizing enterocolitis in newborn rats[18]. Similar findings in other organs point to an overall protective effect.

In a randomized placebo controlled trial involving 53 adults after acute stroke it has been shown that EPO in high dose (100’000 U in three doses) is safe and beneficial[25]. Patients who received EPO had better clinical outcome and smaller lesions in the MRI one month after stroke.

In an ongoing trial in asphyxiated term newborns with high dose EPO no safety concerns could be identified up to now, but definitive results are not yet available[9].

EPO has been proposed for neuroprotection in preterm infants[26] but to our knowledge no clinical studies are currently under way.

2.2.2 Pharmacokinetics of EPO

Studies in rats and sheep have shown greater clearances, shorter half-lives, shorter residence times and greater distribution volumes after i.v. bolus of EPO in fetuses and newborns compared to adults[27]. Consequently the recommended dose per kg body weight for prevention of anaemia is higher in preterm infants than in adults.

Single dose pharmacokinetics of EPO have been studied in preterm infants after intravenous and subcutaneous administration[28, 29]. Mean (±SD) volume of distribution was 300 (±72) ml/kg, total body clearance 27 (±9) ml/h*kg, half life 8 (±3) hours and maximum serum concentration 711 (±166) U/l after 200 U/kg i.v. In the stroke trial serum levels rose to 5649 (±903) mU/ml after the third dose of 33’000 U i.v. resulting in cerebrospinal fluid concentrations of 17.1 (±5.6) mU/ml which is 60 to 100 times that of non-treated patients.

To be neuroprotective EPO must cross the blood-brain barrier. This has been shown to occur in rats after experimental brain injury[7] and in sheep and nonhuman primates[8]. Juul et al showed a huge increase of endogenous EPO after asphyxia in the cerebrospinal fluid in human newborns (225±155 mU/ml, range 7 to 2350 mU/ml) compared to control infants (6±1 mU/ml)[30].

The minimal effective dose for neuroprotection in preterm infants is not known. In the adult stroke trial 100’000 IU EPO divided in three doses were given intravenously and were safe and effective[25]. In rats 5’000 U/kg had the same effect as 10’000 U/kg, both were well tolerated (REF Wang 2004). We consider a dose of 3’000 U/kg as sufficient to achieve a CSF concentration of 5U/ml assuming a Serum/CSF ratio of 100 to 1000.

Recently new derivatives of EPO have been engineered. Darbepoetin-α has 3-fold longer half-life than epoetin-α[31]. Carbamylated EPO[32] and asialeryhtropoietin[33] are tissue protective but not erythropoietic. These substances are not yet established in clinical routine and their specific properties, e.g. longer survival and lack of erythropoietic effect, have no advantage over the classical EPO in preterm infants.

2.2.3 Target Population

Very preterm infants often have significant delay in mental and physical development assessed at 24 months corrected age (Figure 1)[1,2,3,4]. Known causal factors are chorioamnionitis and hypoxic-ischaemic events before, during and after birth. Even in preterm infants without documented perinatal risk factors cerebral development may be impaired.

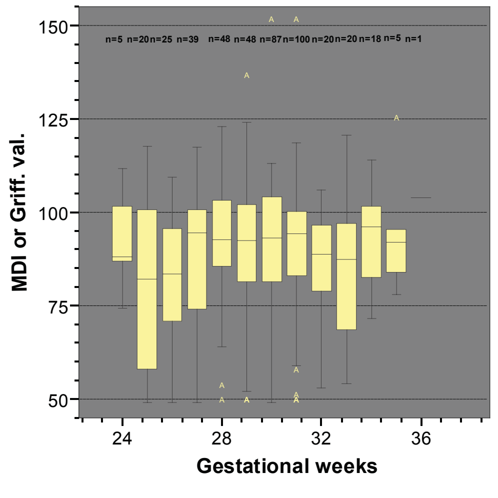

Figure 1:

Developmental outcome in 435 preterm infants born in 2000 in Switzerland before 32 gestational weeks at 24 months corrected for prematurity. Mean Developmental Quotient for infants born at term is 100.

(Swiss Neonatal Network)

Furthermore very preterm infants are at high risk of developing intraventricular haemorrhage, white matter injury, retinopathy, persistent ductus arteriosus, bronchopulmonary dysplasia, septicaemia and necrotising enterocolitis.

3 Study Objectives

3.1 Hypothesis

Early administration of EPO in very preterm infants reduces perinatal injury to the brain, eye, lung and gut and improves neurodevelopmental outcome at 24 months corrected age.

Primary Objective

To determine whether cerebral outcome is improved if infants born between 26 $^{0}/_{7}$ and 31 $^{6}/_{7}$ gestational weeks at birth receive erythropoietin in high dose in the first three days after birth.

Definition of the Primary Objective

Primary endpoint: At 24 months of age corrected for prematurity Bayley Scales of Infant Development (BSID-II) and the presence or absence of impairment of motor function (cerebral palsy) and neurosensory function (blindness or deafness) will be assessed.

Secondary Objectives

To determine whether early administration of erythropoietin alters the incidence of complications typically associated with preterm birth, i.e. mortality, septicaemia, necrotising enterocolitis, bronchopulmonary dysplasia (oxygen dependency at 36 weeks postmenstrual age), retinopathy, intracranial haemorrhage, white matter disease (periventricular leucomalacia), hypertension, growth failure or cerebral palsy and brainvolume at 40 weeks postmenstrual age (MRI).

To evaluate cognitive and fine motor function, vision, hearing and behaviour at five years corrected.
An ancillary economic evaluation is planned in a separate study.

4. Study design

Design

This randomized trial will be double-masked and placebo-controlled. It will use established definitions for tests accepted and introduced within the Swiss Neonatal Network & follow-up group.

Randomisation

EPO as a verum is compared to NaCl 0.9% as a placebo 1:1.
Randomisation lists for block randomisation with variable block length will be created by Dr. A. Tschopp and provided only to the Kantonsapotheke Zurich (Dr. D. Fetz) involved in production of the medication.

4.3 Study duration

The study is scheduled to begin on 01 January 2006. The in-hospital phase of the study will last minimum 8 weeks, maximum 14 weeks (until 40 completed weeks of gestation). All patients will

be followed up for developmental outcome at 24 months after term and at five years. Therefore the whole study will last five years for each participating child.

5. Study Population

Recruitment

To determine whether early administration of EPO alters the incidence and severity of complications typically associated with preterm birth in infants born between 26 $^{0}/_{7}$ and 31 $^{6}/_{7}$ gestational weeks at birth patients were examined to their suitability for this study in terms of the inclusion and exclusion criteria.

5.2 Inclusion criteria

1. Infants born between 26 $^{0}/_{7}$ and 31 $^{6}/_{7}$ gestational weeks
2. Postnatal age less than 3 hours
3. Informed parental consent (preferably obtained before birth)

5.3 Exclusion criteria

1. Genetically defined syndrome
2. Severe congenital malformation adversely affecting life expectancy
3. Severe congenital malformation adversely affecting neurodevelopment
4. A priory palliative care
5. Intracranial haemorrhage grade 2 or more detected before dose 3 of EPO

6. Study medication

6.1 Investigational drug

EPO besides promoting red cell formation has been shown to be protective against hypoxic-ischaemic and inflammatory injuries in a broad range of tissues and organs. Especially a protective effect on brain, retina and bowel could be shown in animal models and first human studies. Several mechanisms explaining this activity have been recognized: EPO inhibits glutamate release in the brain, modulates intracellular calcium metabolism, induces the generation of anti-apoptotic factors, reduces inflammation, decreases nitric oxide–mediated injury, and has direct antioxidant effects.

Very preterm infants may suffer from a variety of short-term complications and long-term sequelae of premature birth. The most critical period are the first days after birth and inflammatory changes as consequence of hypoxia-ischaemia or infection seems to have a major impact on short-term as well as permanent damage.

Because EPO have been shown to influence several mechanisms associated with these short-term and long-term complications of prematurity and furthermore has been shown to have a positive effect even post hoc, i.e. if given within a period of hours after an hypoxic-ischaemic insult, EPO may ameliorate the damage in very premature infants.

EPO has been used widely for several weeks in preterm infants to prevent anemia and is well tolerated. No short and long term adverse effects have been documented with EPO treatment in preterm infants.

Endogenous EPO production may contaminate the difference between EPO and placebo. However measured serum concentrations in non-newborn infants are 100 times lower than those expected after intravenous administration of 25 µg per kg[30]. Some center give EPO routinely in anemic infants to reduce the need for blood transfusion. This low dose treatment is at the discretion of the physician after day 7 as it has been shown not to affect neurodevelopmental outcome.

Blinding, Packaging, Storage Instructions, Labelling, Shelf Life

EPO or an equivalent volume of normal saline (NaCl 0,9%) placebo will be administered intravenously before 3 hours, at 12-18 and at 36-42 hours after birth. A single dose consists of 25 µg (3000 IU) human erythropoietin per kilogram body weight dissolved in 1 ml aqua dest. 1 ml per kilogram birth weight (not the actual weight) will be given intravenously within 3 minutes. The maximal dose is 37.5 µg (4500 IU) for infants weighing 1.5 kilograms or more. The packaging, blinding, labelling and storage of the medicine at the pharmacy conforms to the HMG, according to a randomisation list that is only known to the pharmacy.

The individual vials will be allocated unequivocally by their labelling (centre, patient number).
Upon request, 1 container per patient will be supplied, allowing 3 days of treatment. Adequate refrigeration (+2 to + 8°C) must be provided during storage at the individual centres, as well as during shipment to the centres. The filled containers have a shelf life of at least 1 week in unopened condition, and are microbiologically innocuous. Unused samples must be stored by the individual centres until the end of the study, and then discarded at the centre in accordance with the monitors' instructions. Disposal of samples must be documented in a disposal protocol (included in the investigator's file).

Undesirable effects

Serious, life-threatening side effects have not been observed to date, neither within the scope of approved application of Epo in adults, nor within the established therapy in preterm infants to prevent anaemia.

During and at least 24 hours after injection heart rate, arterial saturation (pulse oximetry) or transcutaneous pO_2 and (arterial) blood pressure will be monitored and the infant will be observed clinically.

Haematological examination including haematocrit, haemoglobin, red and white cell count and thrombocytes will be done at day 7 to 10.

Description of Verum Treatment

Recombinant human EPO is administered once daily. A single dose consists of 25 µg (3000 IU) human erythropoietin per kilogram body weight dissolved in 1 ml aqua dest. 1 ml per kilogram birth weight (not the actual weight) will be given intravenously within 3 minutes. The maximal dose is 37.5 µg (4500 IU) for infants weighing 1.5 kilograms or more.

6.5Description of Placebo Treatment

NaCl 0,9% is administered once daily. A single dose consists of 1 ml NaCl 0,9% per kilogram body weight. 1 ml per kilogram birth weight (not the actual weight) will be given intravenously within 3 minutes.

Allowed/Disallowed Concomitant Medication

Any concomitant medication that is medically indicated for a patient will be allowed within the study.

Reference Sample

A reference sample will be stored by the pharmacy in line with the safety measures demanded until the end of the study.

7. Study Procedures and Examination Methods

Inclusion in the Study, Beginning of Study

All patients will be assessed with regard to inclusion/exclusion criteria (sections 5.2 and 5.3). If the criteria are met, the patient shall be included in the study after parental consent has been obtained, and treatment shall immediately be initiated.
The study medication will be obtained from the responsible pharmacy (cf. 6.2). It will be supplied on request. The study centres will be randomised separately.

7.2Study-related Procedures

Within the study, the following tests will be performed routinely (medically indicated tests and examinations):

1. Minimal electronic monitoring includes heart rate (or pulse rate) with recommended alarm limits at 90 and 180 beats per minute, aterial saturation (pulse oximetry) with recommended alarm limits at 85% and 95%.
2. Arterial blood pressure will be monitored continuously if there is an indwelling arterial line and intermittently by oscillometric measurement at least every 8 hours during the first 7 days.
3. In selected infants cerebral perfusion and oxygenation will be monitored with near infrared spectroscopy (NIRS) 10 min before until 60 minutes after first epo/placebo dose (optional).
4. Serial cerebral ultrasound assessment will be carried out on day 1 and 7 to 10 and then every 14 days until 36 $^{0}/_{7}$ weeks postmenstrual age (or at discharge if discharged before) to detect intracranial haemorrhage graded after Papile[43], ventricular dilatation[44] and white matter disease (periventricular echodensities at 7 to 10 days and periventricular leukomalacia at 36 postmenstrual weeks). The final cerebral ultrasound includes the worst findings in that hospitalisation.
5. Both eyes are examined by an ophthalmologist to detect retinopathy of prematurity (ROP). The severity of ROP will be graded according to the international classification of ROP[45].
6. Necrotising enterocolitis is diagnosed by clinical signs (abdominal distension, bilious aspirates, and/or bloody stools) and either pneumatosis intestinalis, hepatobiliary gas, or free intraperitoneal air on abdominal x-ray or at laparotomy. Severity of enterocolitis will be graded according to presence and severity of complications.
7. Septicaemia is diagnosed by clear clinical, radiological, or histological evidence of infection and at least one microbiologically relevant positive blood culture.
8. Persistent ductus arteriosus (PDA) is diagnosed clinically or with echocardiography and documented only if drug treatment (indomethacin or ibuprofen) or surgical ligation is required.
9. Weight and head circumference are recorded at birth, at 7 to 10 days, at 36 $^{0}/_{7}$ postmenstrual weeks, at 24 months and 5 years corrected age.
10. Optional MRI examination of the head will be performed at the age of 40 (+/- 4 weeks) postmenstrual weeks. Data will be analysed by Prof. P. Hüppi, Geneva.

7.3 Study-related Tests and Examinations

To determine whether early administration of EPO alters the incidence and severity of complications typically associated with preterm birth in infants born between 26 $^{0}/_{7}$ and 31 $^{6}/_{7}$ gestational weeks at birth. Specifically severity of intracranial haemorrhage with its sequelae, white matter disease (periventricular leukomalacia), retinopathy and necrotising enterocolitis and its complications will be monitored, respectively. Further complications such as bronchopulmonary dysplasia (oxygen dependency at 36 weeks postmenstrual age), septicaemia and persistent ductus arteriosus (PDA) will be looked after as well.

Within the study, the following tests will be performed in addition to the medically indicated tests and examinations (cf. 10.2):

1. Placental histology will identify prenatal risks such as amnion infection and placental malfunction. Adaptation will be documented with the APGAR score at 1, 5 and 10 minutes.
2. A sample of umbilical cord serum will we stored to determine the concentration of endogeonous EPO (Prof. C. Dame, Berlin)
3. The Clinical Risk Index for Babies (CRIB). The CRIB is a simple tool for assessing neonatal risk based on the variables gestational age, birth weight, congenital malformations, lowest and highest appropriate FiO2 and worst base deficit within the first 12 hours of life[42].

7.4 Magnetic resonance imaging

In addition to the original protocol MRI imaging at 40 weeks gestation shall be used to quantify the effect of EPO on brain growth, specifically on the cerebral white matter (WM).

7.4.1. Rationale for using MRI

Recent advances in **magnetic resonance (MR) techniques** show great potential to expand our understanding of brain development and brain injury in the living infant. Conventional T1 and T2 weighted imaging can show signal abnormality in the periventricular WM that are different from the cystic lesions detected by ultrasound. The typical conventional MR imaging pattern in the subacute phase consists of punctate periventricular areas of T1 signal hyperintensities and T2 signal hypointensities [1]. In several recent studies on preterm infants brain diffuse excessive high signal intensity (DEHSI) in the cerebral WM on T2-weighted imaging was reported to be present in up to 40 to 75% of low birthweight preterm infants imaged at term [2,3].

The MR techniques provide a unique ability to measure the impact of EPO on subsequent brain development. Specifically, the development of 3D **volumetric MRI** techniques allows quantification of the absolute volume of cerebral WM, both myelinated and unmyelinated components, as well as cortical gray matter and basal structures such as the striatum.

With the advent of improved imaging techniques the hypothesis of a sensitive period of brain development in the last trimester of gestation becomes increasingly testable. To study effects of extrauterine life on brain development a group of healthy preterm infants (<32 weeks at birth) and a group of healthy fullterm infants underwent conventional MR-imaging at 40-41 weeks with semi-quantitative analysis. The preterm infants at term showed significantly less gray/WM differentiation and less myelination. They also showed poorer neurobehavioral performance[4].

Volumetric 3D-MRI and Segmentation Image Processing: Acquisition of Normative Data in Preterm Infants

In a subsequent study the group developed advanced volumetric 3D-MRI techniques to focus on more detail of brain development in the period from 28 weeks to term and elaborate on the processes likely to underlie the preterm/fullterm differences found at term described in the earlier study. Post acquisition image processing with tissue segmentation algorithms were developed to quantitate total brain volume and absolute volumes of gray and WM in premature and term infants[5]. While total brain tissue volume exclusive of cerebrospinal fluid (CSF) was found to increase in the period of 29 to 41 weeks, the principal determinant was found to be an increase in gray matter volume (GM) from 35 to 50% that related to a fourfold increase in cortical GM volume rather than in subcortical GM volume. This increase is thought to primarily relate to cortical differentiation rather than to an increase in the total number of neurons. The cortical GM volume increase was found to be associated with significant increase in surface area and gyral development[5]. Furthermore we have been able to distinguish and quantitate the absolute volumes of both myelinated and unmyelinated WM (WM). Prior to 36 weeks of gestation, unmyelinated WM was the dominant type of brain tissue. However, starting at 35 weeks and up to 41 weeks an almost 5-fold increase in absolute volume of myelinated WM was observed. This MR technique gives us the ability to measure quantitatively the impact both on myelination and cortical development of insults at the critical period of brain development[6].

Volumetric MRI in Preterm Infants with WM Injury Studied at Term

In a first study to apply these techniques to study effects of brain injury 3-D MRI volumetric techniques were used to evaluate the effect on subsequent brain development of early WM injury in premature infants. Studies were done at 40 weeks postconceptional age in a total of 34 infants. In the premature infants with preceding WM injury, the volume of total gray matter, cortical gray matter and myelinated WM at term was significantly lower than in both the premature infants without prior WM injury and the infants born at term[7]. These data support the hypothesis that injury to the developing WM during the critical period of WM maturation will result in impairment not only of the very early phases of myelination, but also will alter subsequent cortical development, detectable by volumetric MRI at term.

Table. Quantitative 3D-MRI Volumes of Cerebral Tissues at Term in Premature Infants with Evidence of PVL, Premature Infants with no Evidence of PVL, and Healthy Term Infants

	Premature with PVL, at Term (n = 10)	Premature with No PVL, at Term (n = 10)	Normal Term (n = 14)	*p* (ANOVA, Pairwise Multiple Comparisons)
Cerebral cortical gray matter	157.5 ± 41.5	211.7 ± 25.4	218.8 ± 21.3	<0.001
Basal ganglia/thalamus	22.5 ± 7.6	29.7 ± 14.2	24.7 ± 9.1	NS
Total gray matter	180.1 ± 49.5	241 ± 39.6	243 ± 30.4	0.001
Myelinated white matter	14.5 ± 4.6	23.1 ± 6.9	27.6 ± 10.3	0.002
Unmyelinated white matter	178.3 ± 33.9	212.8 ± 28.4	200.3 ± 32.9	NS
Total white matter	192.8 ± 38.9	235.9 ± 35.3	227.9 ± 43.7	NS
CSF	64.5 ± 15.2	52.0 ± 24.1	32.9 ± 13.5	<0.001

Data are volumes in milliliters and are mean ± SD values.

3D-MRI = three-dimensional magnetic resonance imaging; PVL = periventricular leukomalacia; ANOVA = analysis of variance; CSF = cerebrospinal fluid.

Diffusion Weighted Imaging (DWI) in newborns

The DWI technique measures the diffusivity of brain water and is quantitatively expressed as the apparent diffusion coefficient (ADC). Experience with the DWI technique has been confined largely to the study of stroke, primarily in adults, but also to some extent in term newborns [8]. ADC

changes precede the appearance of abnormalities on conventional T2-weighted MRI for ischemic injury [7]. However, until recently, normative data had been unavailable for the premature brain, limiting the ability to identify ischemic injury in the newborn by this technique.

To determine the normal developmental changes of the ADC in cerebral WM, DWI studies in preterm and term newborns (GA from 28 to 40 weeks) were performed within the first two weeks of life. The data showed important changes in the quantitative measurements of water diffusion in the developing brain. Specifically, there was a marked decline in the ADC values in the central WM over this gestational age range, indicative of a progressive decrease in water diffusion. These findings provided new insights into the implications of changes in ADC values with WM development and will be used as a baseline for the data collected in the current study [9].

To provide insight into the nature of the microstructural developmental events associated with the decrease of the ADC described above, diffusion tensor analysis can be used to determine diffusion anisotropy, a measure of the directionality of diffusion in cerebral WM. In this study on developmental changes of the DTI parameters relative anisotropy (RA) showed a striking increase in cerebral WM from 28 to 40 weeks [9].

7.4.2 Additional Objectives for the MRI

To determine whether early rhEPO treatment will improve neurostructural development at term assessed as (a) increased myelinated white matter volume (by quantitative volumetric MRI) (b) improved microstructural development (by diffusion tensor MRI) of cerebral white matter and (c) increased cortical and subcortical (basal ganglia) gray matter volume (by quantitative volumetric MRI).

To assess whether modern MR-imaging techniques, e.g. diffusion tensor imaging (DTI), and MR spectroscopy (MRS) can predict neurological abnormalities in preterm infants.

7.4.3 Procedure of MRI examination

Infants will be studied at 40 weeks gestation (near the expected term) during natural sleep at the Center for magnetic resonance, Children's Hospital Zurich and Geneva. The parents will bring their infants to the center, feed them and let them fall asleep. The infants will then be fixated on a table with heated mattress and brought to the magnet. If they wake up, they will get a sweet solution with a pacifier. If they can not be calmed, the study will be terminated.

7.4.4 Analysis of MRI data

In Zurich MRI data will be stored on CD and sent to Geneva by B. Koller. Volumetric assessment to demonstrate the effect of EPO (Objective 1) will be done by P. Hüppi, Geneva. Sophisticated DTI and fibre tracking methods will be used by E. Martin to predict neurological outcome (objective 2).

7.4.5 Information of parents

The parents will be informed about the planned MRI examination before the infant is discharged home and have to give written consent. If there is a medical indication for the MRI examination (for example pathologigal cerebral ultrasound finding) they will only have to agree that the results are also analysed for the Epo-trial.

If the parents do not want an MRI examination, the infant will stay in the study cohort as the main outcome is developmental status at 24 months corrected for prematurity.

The results of the MRI-analysis will be explained to the parents by Dr. Bea Latal or Prof. Hüppi

7.5 Individual Withdrawal Criteria

A patient will be withdrawn from the study when one of the following criteria is fulfilled:

1. violation of the study protocol
2. intolerable side effects
3. any conditions that might jeopardize the patient's health if they were to continue in the study

7.6 Individual End of the Study

The end of the study for each patient will be death, discharge home or gestational age of 40 (+4 weeks) completed weeks.

7.7 Patient's consent, information and data protection declaration

As this study involves paediatric patients, the parents will receive information on the study, and give their informed consent for its implementation as the child's legal agents. A data protection declaration will have to be provided for them to sign.

7.8 Follow up Study

The follow up study should be based on the assessment of developmental function and handicaps at 24 months as well as 5 years corrected age.

Assessment at 24 months corrected age uses the Bayley Scales of Infant Development (BSID-II). At the same examination the presence or absence of impairment of motor function (cerebral palsy) and neurosensory function (blindness or deafness) will be assessed.

The BSID-II is well standardised, reliable and valid[38], and one of the most widely used psychometric tests. The MDI scores have a mean of 100 and a SD of 15. This test will allow to detect subtle deviations in cognition. A previously used classification scheme[39] will be used to code the severity of disability (nil, mild, moderate, severe).

Gross motor function will be classified as described by Palisano[40] and used in the ongoing caffeine trial[41]. Blindness will be defined as corrected visual acuity of less than 20/200. Deafness will be defined as hearing loss requiring amplification.

BSID-II has been used by several centres since many years and has been accepted as the standard in the Swiss Neonatal Network together with a neurological examination focusing on gross motor function. All assessments must be performed by trained and experienced examiners.

At 5 years corrected age cognitive development will be assessed with a reliable and validated test (K-ABC II). A standardised neurological, visual and hearing examination will be performed. These tests will be done by trained psychologists or physicians in each centre. The parents will fill in a questionnaire on the child health status and behaviour.

Socioeconomic status will be calculated according to Largo[46] by means of a six-point score of both paternal occupation and maternal education. The lowest possible SES score is 1, the highest 12. Socioeconomic status has been shown to affect cognitive development and therefore is an important confounder, which should be balanced in the EPO group and the placebo group.

The International Classification of Impairments, Disabilities, and Handicaps[47] distinguishes between consequences of diseases and disorders at the level of the body (impairment), the person (disability or limitation of functional activity), and the person as a social being (handicap or limitation of participation in society). In the 24 months assessment we will focus on ”disability”, in the 5 years assessment we will also include ”handicap”.

7.9 Randomisation and Deblinding

The study is randomised on a double-blind basis. This means that the attending investigators do not know whether they are using the placebo or the verum. Randomisation is performed on the basis of randomisation lists (see 4.2).

In the event that deblinding should be required subsequent to an individual patient's withdrawal from the study, this will be possible by immediate notification of the pharmacy responsible for the study centres, with specification of the centre and randomisation number, within 24 hours.

In the event of deblinding, the Principal Investigator has to be notified within a period of 24 hours. The PI will then take charge of further reporting as required by the GCP guidelines.

8. Methods for Assessing Efficacy and Safety

Appraisal of the Risk-to-Benefit Ratio

In the many EPO trials both in preterm infants and in adults EPO was well tolerated. Adverse effects described in adults are arterial hypertension, polycytaemia, thrombosis and antibody formation against EPO-receptors. As preterm infants tend to have low blood pressure, anaemia and delayed coagulation these side effect of EPO may even be beneficial. Antibody formation occurred in kidney patients after long-term subcutaneous administration of EPO[34,35] and therefore is very unlikely to be a problem in preterm infants after three intravenous injections. Carcinogenicity finally was observed exclusively in cancer patients who received EPO to treat anaemia, which is hardly relevant for preterm infants[36].

One retrospective study reports a higher incidence of retinopathy of prematurity in preterm infants treated over 6 weeks with EPO to prevent anaemia compared with controls[49]. This finding could not be confirmed in other prospective randomised controlled trials[23,24,50].

In summary EPO, is considered to be safe. To eliminate the minimal doubts, during this trial infant will be monitored continuously and all adverse events noted and sent to the sponsor at day 10. The safety board will continuously monitor the adverse events and stop the trial if significantly more complications ($p<0.01$) occur in the verum group.

The nature and extent of the therapeutic interventions employed within the study described here do not go beyond what is therapeutically required for these patients.

Methods for Assessing Efficacy

Primary endpoint: At 24 months of age corrected for prematurity Bayley Scales of Infant Development (BSID-II) and the presence or absence of impairment of motor function (cerebral palsy) and neurosensory function (blindness or deafness) will be assessed.

Secondary endpoints:

- ultrasound of the brain will demonstrate if Epo reduces the incidence of brain damage, specified as intracranial haemorrhage graded after Papile[43], ventricular dilatation[44] and white matter disease (periventricular echodensities).
- the occurrence of mortality, septicaemia, necrotising enterocolitis, bronchopulmonary dysplasia (oxygen dependency at 36 weeks postmenstrual age) and retinopathy will be documented in each infant.

- MRI at 40 weeks to assess (a) increased myelinated white matter volume (by quantitative volumetric MRI) (b) microstructural development (by diffusion tensor MRI) of cerebral white matter and (c) cortical and subcortical (basal ganglia) gray matter volume (by quantitative volumetric MRI). This examination will only be optional.

Adverse Events (AE), Definition

Adverse events are taken to be any general lack of well-being, symptoms of illness, laboratory parameters outside the normal range and other impairments observed within the context of a clinical study. This applies regardless of any causal connection with the investigational study drug administered.

Any event not attributable to the category „serious“ (see section 8.4) is considered an adverse event (AE).

8.4 Serious Adverse Events (SAE), Definition

A serious adverse event (SAE) is any event that is life-threatening or fatal

- results in significant or permanent disability results in a tumour disease or malformation
- meets any other comparable criteria

Such events are considered serious, regardless of any causal connection with the investigational study drug administered.

SAE, AE: Evaluation, Assessment, Documentation

Adverse events (AE) and serious adverse events (SAE) are documented in the data collection form.

For each adverse event, the investigator will assess the degree of severity, the intensity, and the connection with investigational study drug; the onset and the end of the event, as well as the counteractive measures taken and the further course of the event, will be documented by the investigator.

Serious adverse events (SAE) have to be reported to the Sponsor within 24 hours by the PI; the Sponsor will then take charge of further reporting procedures as required by the HMG and VKlin. Such a report to the Sponsor can be done by fax (01 255 44 42), telephone (01 255 53 41 or 01 255 53 08) or by e-mail (buh@usz.ch). For documentation purposes, a special form is provided along with the data collection form.

8.6 Premature Termination of the Clinical Trial

For the patients’ benefit and in their best interest, the Principal Investigator (PI) or an individual investigator may terminate the study within their area of responsibility at any time. In this case, the appropriate Ethics committee will be notified immediately.

9. Information on Statistics, Evaluation

9.1 Number of Cases, Statistical Considerations

Diagram: theoretical patient recruitment and progress

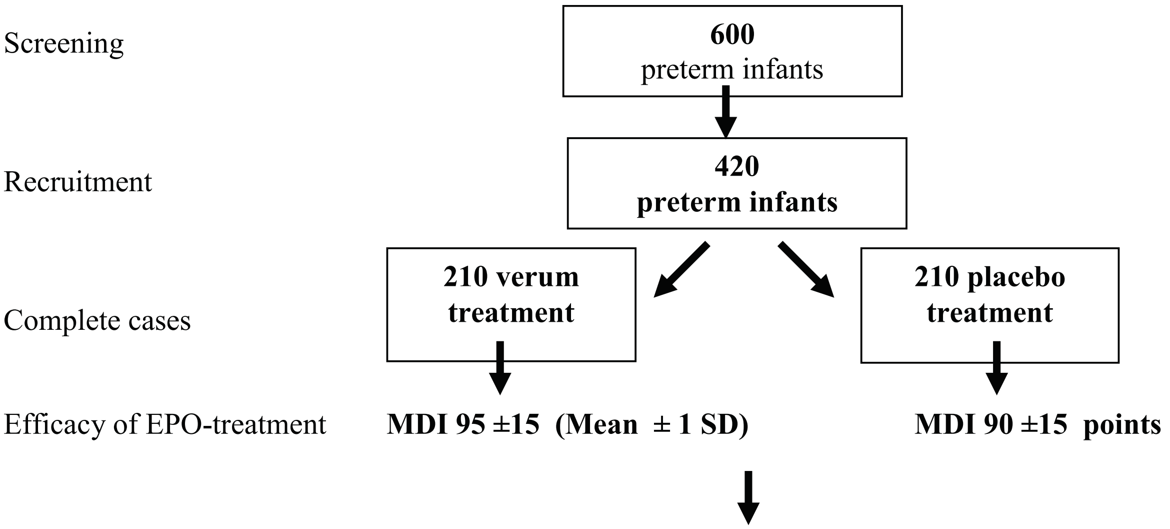

Assumption two-sided alpha 0.05, power (1-beta) 0,8

Hypothesis **$MDI_{verum} - MDI_{placebo} > 5$ points (0.3 SD)**

Number needed per group 176 (+20% to compensate drop-outs)

Biometric Planning and Data Evaluation

Analysis of the data will be done after inputting all the data, after plausibility checks and termination of monitoring (data lock), at the earliest from spring 2009 and no later than spring 2013.

First the success of randomisation will be documented by checking the treatment groups for uniform composition with regard to age, sex, body weight, and degree of prematurity. For this purpose, a table will be created with measures of location and scatter, depending on the type of scale. Subsequently, the parameters needed to test the hypothesis on the primary objective will be estimated as described above. It will be attempted to take into account the influence of known risk factors for brain damage, as well. Next, the secondary objectives will be described in their dependence on the influencing and disturbance variables. Simple models of variance analysis will be used to describe the central tendency. 99% confidence intervals will be given for effects, so that a high confidence level can also be obtained when interpreting several effects. The secondary target variables will be transformed where necessary (e.g. take logarithms of concentrations) so that the assumptions of the linear model are justified. The results will be transformed back onto the original scale. Example: geometric mean as an estimate of the median and coefficient of variation as an indication of relative error.

Kaplan-Meier curves will be provided for durations of stay. For binomially distributed target variables, a logic regression will be applied.

Interim Analysis

After treatment of the first 45 patients, the sponsor will draw up a report on the adverse events and the serious adverse events plus the group sizes, which he will then discuss with the safety board. The report, as well as the minutes of the discussion and any potential consequences from this, will be communicated in writing to all study centres.

10.Data Collection Forms, Data Management

Data Collection Forms

All the relevant patient data for the study will be recorded in anonymous format on a Case Report Form (CRF). The following data will be documented: The patient's randomisation number study-specific procedures (administration of the investigational drug, serum sampling), inclusion and exclusion criteria, demographic data, medical history and concomitant treatments, findings copied from the patient's file, follow-up observation of the period of ventilation and the planned length of stay.

Data will be entered on-line via a protected access to a data bank hosted at the CTU Zurich. Plausibility checks will be performed continuously and data entry will be checked by a monitor. Query forms integrated in the data management software will be used to explain and correct with the investigating physician.

How will individual findings be collected and documented?

Data will be entered on-line from the patients' files (original data, source data) in each participating center. The following data will be collected on the basis of the patient file, depending on ventilation time:

- Apgar and CRIB score
- Ventilation (ventilation route, ventilation type, FiO_2,)
- Blood gas analysis (pH, pO_2, pCO_2, BE, HCO_3^-, SaO_2)
- Blood counts (haemoglobin, leukocytes, thrombocytes) clinical chemistry: Na, K, creatinine, urea
- Pathogen detection until 36 week of gestation age
- Weight, head circumference at birth and at day 7-10 and 36 $^0/_7$ postmenstrual weeks

Additionally, the following materials will be forwarded to the appropriate addresses for testing:

- Cord serum (EPO concentration)
- Continous NIRS monitoring data before and after first dose for assessment of changes in tissue oxygenation index (only selected infants in Zurich).
- MRI at 40 weeks of gestation (optional)

Patient Identification List

In the CRFs, all patients are anonymised by means of a patient number; to be identified only via the patient identification list. Each centre's patient identification list will be kept exclusively by the investigating physician in the investigating physician's file.

Filing of Study Records

Upon completion of the study, the patient identification list will be filed by the individual investigator for a period of at least 15 years. Likewise, the responsible investigators at the individual study centres will store the Case Report Forms (CRF), as well as the investigator's file containing all pertinent documents, for a minimum period of 15 years after completion of the study.

The trial master file, which is the central file that also contains all pertinent study documents, will be retained by the principal investigator (PI) after the completion of the study for a period of at least 15 years, as will the original findings of the study-specific examinations and the results from the CRFs collated by the monitor.

11. Ethical Issues, Data Protection, Quality Assurance, Insurance

ICH/GCP Guidelines, the Helsinki Declaration, Legal Provisions

The Helsinki Declaration shall be applied to the clinical trial, as well as Good Clinical Practice (GCP) for conducting clinical trials of medicinal products within the European Community, in its current version.

Assessment of the Protocol by the Responsible Ethics Committee

The protocol (version 13 dated 1 June 2005, revised version 15 dated 15 October 2005 and version 16 dated 30 June 2008) have been approved by the Ethics Committee of the Children's Hospital Zurich and the "Kantonale Ethikkommission des Kantons Zürich".
In all further proceedings, the investigator at each participating centre will have to submit the protocol to the respective local Independent Ethics Committee.

Handling of Additions/Changes to the Protocol

With a view to ensuring comparable conditions and faultless data evaluation, changes to the protocol are not planned. In some exceptional cases, however, this could be necessary. Any additions and changes made to the protocol have to be submitted to the appropriate Ethics Committee and authorities for review. Changes to protocol procedures (amendments) require a specification of reasons and must be signed by an authorised signatory for the respective protocol; the amendments are then considered part of the protocol. Substantial changes, in particular with regard to patients' health interests, require a new vote from the Ethics Committee.

Information for Parents

Parents will be given printed information that will be discussed with them.

Data Protection Statement

The written permission for use of the personal and study-related data and passing it on in anonymous form will be obtained before a patient is included. The patients' parents have to give their consent on behalf of their child for this data to be used within a scientific study. If no such consent is given, the data must not be used.
Only those persons involved in the clinical trial will have access to the personal data and to the patient identification list on a need-to-know basis.

Monitoring, Inspections

Monitoring is employed primarily for the subjects' safety, as well as for quality assurance of medical procedures. The centres will be visited by members of the data safety and monitoring board on a regular basis. Under adherence to the laws on data protection, the investigator's files, data collection forms, and original documents have to be made available to the monitor. The investigators discuss the course of the study with the monitor in appropriate form.
Trial institutions, facilities, laboratories, all data (including raw data) and data collection forms must always be available for inspection by an authority.

Insurance

Insurance is covered by *Haftpflichtversicherung für den Kanton Zürich betreffend das UniversitätsSpital Zürich* (Vertrags-Nr. 8.404.911).
So as not to forfeit their insurance cover, the patients themselves must comply with the following conditions: Any deterioration in the patient's state of health that may have occurred as a result of the clinical trial must be reported immediately to the investigator, so he or she can notify the insurance company. The patient and his or her parents must take any appropriate measures that may help to determine the cause or the extent of damage, and to minimise the damage. In the event of a patient's death, the insurer must be notified immediately. The patient must not be involved in any other clinical trial during the course of this trial, nor within a period of 30 days prior to its beginning or 30 days after its completion.
A copy of the insurance certificate will be placed in the investigating physician's file.

General Provisions, Agreements, Organisational Procedures

12.1 Reports to Authorities

This protocol has been sent to the Federal Institute for Drugs and Medical Devices (Swissmedic) and the study number StV-36/04 has been assigned. Final approval is still pending. Reporting to the appropriate local supervising authorities for each centre will be effected individually by the responsible investigators.

Final Report

The Final Report for the study will be compiled by the Principal Investigator within a period of 90 days upon completion of the study, and forwarded to the centres, to the Ethics Committees, and to authorities. Furthermore, the sponsor shall undertake to submit the Final Report for publication as soon as possible.

12.3 Publications

Publications will be created by the Study Principal (study authors). A report on the primary and secondary endpoints by treatment will be submitted for publication during 2006, an attempt that on refusal will be repeated several times with journals of different impacts. The list of authors will include between one and three treating physicians per centre (depending on the individual requirements of medical journals). The order is defined as follows: 1. Zürich (Bucher), 2. investigators of each center in order of number of infants included in the study.

The results of the MRI study will be published separately by Hueppi and other authors according to their individual contributions.

With regard to publications, the principles of data protection must be observed for patient data as well as for data pertaining to participating physicians.

13. Literature

1. Bucher HU, Ochsner Y, Fauchere JC. Two years outcome of very pre-term and very low birthweight infants in Switzerland. Swiss Med Wkly 2003; 133:93-9.
2. Bucher HU, Killer C, Ochsner Y, Vaihinger S, Fauchere JC. Growth, developmental milestones and health problems in the first 2 years in very preterm infants compared with term infants: a population based study. Eur J Pediatr 2002; 161:151-6.
3. Vohr BR, Wright LL, Dusick AM, et al. Neurodevelopmental and functional outcomes of extremely low birth weight infants in the National Institute of Child Health and Human Development Neonatal Research Network, 1993-1994. Pediatrics 2000; 105:1216-26.
4. Stoelhorst GM, Rijken M, Martens SE, et al. Developmental outcome at 18 and 24 months of age in very preterm children: a cohort study from 1996 to 1997. Early Hum Dev 2003; 72:83-95.
5. Genc S, Koroglu TF, Genc K. Erythropoietin and the nervous system. Brain Res 2004; 1000:19-31.
6. Marti HH, Bauer C. Expression of erythropoietin and its recetpor in the central nervous system. in Hoke A (Ed). Erythropoietin and the Nervous System 2006, XII, 224 p. ISBN: 0-387-30010-4
7. Brines ML, Ghezzi P, Keenan S, et al. Erythropoietin crosses the blood-brain barrier to protect against experimental brain injury. Proc Natl Acad Sci U S A 2000; 97:10526-31.
8. Juul SE, McPherson RJ, Farrell FX, Jolliffe L, Ness DJ, Gleason CA. Erytropoietin concentrations in cerebrospinal fluid of nonhuman primates and fetal sheep following high-dose recombinant erythropoietin. Biol Neonate 2004; 85:138-44. Epub 2003 Nov 24.
9. Juul S. Erythropoietin in the central nervous system, and its use to prevent hypoxic-ischemic brain damage. Acta Paediatr Suppl 2002; 91:36-42.
10. Aydin A, Genc K, Akhisaroglu M, Yorukoglu K, Gokmen N, Gonullu E. Erythropoietin exerts neuroprotective effect in neonatal rat model of hypoxic-ischemic brain injury. Brain Dev 2003; 25:494-8.
11. Kumral A, Ozer E, Yilmaz O, et al. Neuroprotective effect of erythropoietin on hypoxic-ischemic brain injury in neonatal rats. Biol Neonate 2003; 83:224-8.
12. Junk AK, Mammis A, Savitz SI, et al. Erythropoietin administration protects retinal neurons from acute ischemia-reperfusion injury. Proc Natl Acad Sci U S A 2002; 99:10659-64. Epub 2002 Jul 18.
13. Kumral A, Uysal N, Tugyan K, et al. Erythropoietin improves long-term spatial memory deficits and brain injury following neonatal hypoxia-ischemia in rats. Behav Brain Res 2004; 153:77-86.
14. Siren AL, Knerlich F, Poser W, Gleiter CH, Bruck W, Ehrenreich H. Erythropoietin and erythropoietin receptor in human ischemic/hypoxic brain. Acta Neuropathol (Berl) 2001; 101:271-6.
15. Sun Y, Zhou C, Polk P, Nanda A, Zhang JH. Mechanisms of erythropoietin-induced brain protection in neonatal hypoxia-ischemia rat model. J Cereb Blood Flow Metab 2004; 24:259-70.
16. Spandou E, Soubasi V, Papoutsopoulou S, et al. Erythropoietin prevents hypoxia/ischemia-induced DNA fragmentation in an experimental model of perinatal asphyxia. Neurosci Lett 2004; 366:24-8.
17. Juul SE, Anderson DK, Li Y, Christensen RD. Erythropoietin and erythropoietin receptor in the developing human central nervous system. Pediatr Res 1998; 43:40-9.
18. Kumral A, Baskin H, Duman N, et al. Erythropoietin protects against necrotizing enterocolitis of newborn rats by the inhibiting nitric oxide formation. Biol Neonate 2003; 84:325-9.

19. Halperin DS. Use of recombinant erythropoietin in treatment of the anemia of prematurity. Am J Pediatr Hematol Oncol 1991; 13:351-63.

20. Beck T, Bielenberg GW. Failure of the lipid peroxidation inhibitor U74006F to improve neurological outcome after transient forebrain ischemia in the rat. Brain Res 1990; 532:336-8.

21. Shannon KM, Mentzer WC, Abels RI, et al. Recombinant human erythropoietin in the anemia of prematurity: results of a placebo-controlled pilot study. J Pediatr 1991; 118:949-55.

22. Carnielli V, Montini G, Da Riol R, Dall'Amico R, Cantarutti F. Effect of high doses of human recombinant erythropoietin on the need for blood transfusions in preterm infants. J Pediatr 1992; 121:98-102.

23. Maier RF, Obladen M, Scigalla P, et al. The effect of epoetin beta (recombinant human erythropoietin) on the need for transfusion in very-low-birth-weight infants. European Multicentre Erythropoietin Study Group. N Engl J Med 1994; 330:1173-8.

24. Maier RF, Obladen M, Muller-Hansen I, et al. Early treatment with erythropoietin beta ameliorates anemia and reduces transfusion requirements in infants with birth weights below 1000 g. J Pediatr 2002; 141:8-15.

25. Ehrenreich H, Hasselblatt M, Dembowski C, et al. Erythropoietin therapy for acute stroke is both safe and beneficial. Mol Med 2002; 8:495-505.

26. Strunk T, Hartel C, Schultz C. Does erythropoietin protect the preterm brain? Arch Dis Child Fetal Neonatal Ed 2004; 89:F364-6.

27. Widness JA, Veng-Pedersen P, Modi NB, Schmidt RL, Chestnut DH. Developmental differences in erythropoietin pharmacokinetics: increased clearance and distribution in fetal and neonatal sheep. J Pharmacol Exp Ther 1992; 261:977-84.

28. Brown MS, Jones MA, Ohls RK, Christensen RD. Single-dose pharmacokinetics of recombinant human erythropoietin in preterm infants after intravenous and subcutaneous administration. J Pediatr 1993; 122:655-7.

29. Ohls RK, Veerman MW, Christensen RD. Pharmacokinetics and effectiveness of recombinant erythropoietin administered to preterm infants by continuous infusion in total parenteral nutrition solution. J Pediatr 1996; 128:518-23.

30. Juul SE, Yachnis AT, Rojiani AM, Christensen RD. Immunohistochemical localization of erythropoietin and its receptor in the developing human brain. Pediatr Dev Pathol 1999; 2:148-58.

31. Deicher R, Horl WH. Differentiating factors between erythropoiesis-stimulating agents: a guide to selection for anaemia of chronic kidney disease. Drugs 2004; 64:499-509.

32. Leist M, Ghezzi P, Grasso G, et al. Derivatives of erythropoietin that are tissue protective but not erythropoietic. Science 2004; 305:239-42.

33. Erbayraktar S, Grasso G, Sfacteria A, et al. Asialoerythropoietin is a nonerythropoietic cytokine with broad neuroprotective activity in vivo. Proc Natl Acad Sci U S A 2003; 100:6741-6. Epub 2003 May 13.

34. Schoenholzer C, Keusch G, Nigg L, Robert D, Wauters JP. High prevalence in Switzerland of pure red-cell aplasia due to anti-erythropoietin antibodies in chronic dialysis patients: report of five cases. Nephrol Dial Transplant 2004; 19:2121-5.

35. Casadevall N, Nataf J, Viron B, et al. Pure red-cell aplasia and antierythropoietin antibodies in patients treated with recombinant erythropoietin. N Engl J Med 2002; 346:469-75.

36. Brower V. Erythropoietin may impair, not improve, cancer survival. Nat Med 2003; 9:1439.

37. Bucher HU, Fawer CL, von Kaenel J, Kind C, Moessinger A. Intrauterine and postnatal transfer of high risk newborn infants. Swiss Society of Neonatology. Schweiz Med Wochenschr 1998; 128:1646-53.

38. Bayley N. Bayley Scales of of Infant Development, 1993.

39. The Victorian, Infant. The Victorian Infant Collaborative Study Group: Imroved outcome into the 1990s for infants weighing 500-999g at birth. Arch Dis Child 1997; 77:F91-F94.

40. Palisano R, Rosenbaum P, Walter S, Russell D, Wood E, Galuppi B. Development and reliability of a system to classify gross motor function in children with cerebral palsy. Dev Med Child Neurol 1997; 39:214-23.

41. CAP Trial. Efficacy and safety of methylxanthines in very low birth weight infants. CAP: (Caffeine for Apnea of Prematurity). 2001:1-25.

42. Parry G, Tucker J, Tarnow-Mordi W. CRIB II: an update of the clinical risk index for babies score. Lancet 2003; 361:1789-91.

43. Ment LR, Bada HS, Barnes P, et al. Practice parameter: neuroimaging of the neonate: report of the Quality Standards Subcommittee of the American Academy of Neurology and the Practice Committee of the Child Neurology Society. Neurology 2002; 58:1726-38.

44. Levene MI. Measurement of the growth of the lateral ventricles in preterm infants with real-time ultrasound. Arch Dis Child 1981; 56:900-4.

45. An international classification of retinopathy of prematurity. II. The classification of retinal detachment. The International Committee for the Classification of the Late Stages of Retinopathy of Prematurity. Arch Ophthalmol 1987; 105:906-12.

46. Largo RH, Graf S, Kundu S, Hunziker U, Molinari L. Predicting developmental outcome at school age from infant tests of normal, at-risk and retarded infants. Dev Med Child Neurol 1990; 32:30-45.

47. WHO. International classification of impairments, disabilities and handicaps. A manual of classification relating to the consequences of disease. In: Manual W, ed. Geneva: WHO, 1980.

48. Machin D, Campbel M, Fayers P, A P. Sample size tables for clinical studies, 1997.

49. Romagnoli C, Zecca E, Gallini F, Girlando P, Zuppa AA. Do recombinant human erythropoietin and iron supplementation increase the risk of retinopathy of prematurity? Eur J Pediatr 2000; 159: 627-634.

50. Ohls RK, Ehrenkranz RA, Das A, Dusck AM, Yolton K, Romaon E et al. Neurodevelopmental outcome and growth at 18 to 22 months' corrected age in extremely low birth weight infants treated with early erythropoietin and iron. Pediatrics. 2004; 114: 1287-91.

References for MRI

1. Inder, T.E., et al., White matter injury in the premature infant: a comparison between serial cranial sonographic and MR findings at term. AJNR Am J Neuroradiol, 2003. 24(5): p. 805-9.
2. Huppi, P.S., Advances in postnatal neuroimaging: relevance to pathogenesis and treatment of brain injury. Clin Perinatol, 2002. 29(4): p. 827-56.
3. Maalouf, E.F., et al., Magnetic resonance imaging of the brain in a cohort of extremely preterm infants. J Pediatr, 1999. 135(3): p. 351-7.
4. Huppi, P.S., et al., Structural and neurobehavioral delay in postnatal brain development of preterm infants. Pediatr Res, 1996. 39(5): p. 895-901.
5. Huppi, P.S., et al., Quantitative magnetic resonance imaging of brain development in premature and mature newborns. Ann Neurol, 1998. 43(2): p. 224-35.
6. Tolsa, C.B., et al., Early alteration of structural and functional brain development in premature infants born with intrauterine growth restriction. Pediatr Res, 2004. 56(1): p. 132-8. Epub 2004 May 5.

7. Inder, T.E., et al., Periventricular white matter injury in the premature infant is followed by reduced cerebral cortical gray matter volume at term. Ann Neurol, 1999. 46(5): p. 755-60.
8. Cowan, F.M., et al., Early detection of cerebral infarction and hypoxic ischemic encephalopathy in neonates
9. Huppi, P.S., et al., Microstructural development of human newborn cerebral white matter assessed in vivo by diffusion tensor magnetic resonance imaging. Pediatr Res, 1998. 44(4): p. 584-90

Zürich, September 17, 2008

Prof. H.U. Bucher
