# Supplementary material for: Effect of Early High-Dose Recombinant Human Erythropoietin on Behavior and Quality of Life in Children Aged 5 Years Born Very Preterm: Secondary Analysis of a Randomized Clinical Trial
Source: JAMA Netw Open. 2022 Dec 7;5(12):e2245499. doi: 10.1001/jamanetworkopen.2022.45499 (PMC9856490; doi:10.1001/jamanetworkopen.2022.45499)
Supplement: Supplement 2. — eTable 1. Comparison of Parent-Reported Behavioral Problems According to the SDQ Between Groups at Age 5 Years Restricted to Children Who Completed the Allocated Treatment eTable 2. Comparison of Parent-Reported HRQoL According to the KIDSCREEN-27 Between Groups at Age 5 Years Restricted to Children Who Completed the Allocated Treatment [file jamanetwopen-e2245499-s002.pdf]

Supplemental Online Content

Picotti E, Reinelt T, Koller B, et al; Swiss EPO Neuroprotection Trial Group. Effect of early high-dose recombinant human erythropoietin on behavior and quality of life in children aged 5 years born very preterm: secondary analysis of a randomized clinical trial. *JAMA Netw Open*. 2022;5(12):e2245499. doi:10.1001/jamanetworkopen.2022.45499

eTable 1. Comparison of Parent-Reported Behavioral Problems According to the SDQ Between Groups at Age 5 Years Restricted to Children Who Completed the Allocated Treatment

eTable 2. Comparison of parent-reported HRQoL according to the KIDSCREEN-27 Between Groups at Age 5 Years Restricted to Children Who Completed the Allocated Treatment

This supplemental material has been provided by the authors to give readers additional information about their work.

© 2022 Picotti E et al. *JAMA Network Open.*

eTable 1. Comparison of Parent-Reported Behavioral Problems According to the SDQ Between Groups at Age 5 Years Restricted to Children Who Completed the Allocated Treatment

**Strenght and Difficulties Questionnaire Scales' scores[a]** ***(Cronbach's Alpha)***	**rhEpo N = 140**	**Placebo N = 139**	**Mean Difference (95% CI)**	**OR (95% CI)**	***P*-value**
Emotional problems (.63), Mean (SD)	2.09 (2.01)	1.67 (1.66)	0.43 (0.05 - 0.80)		.06
- at risk score, n (%)	16 (11)	10 (7)		1.68 (0.83 - 3.39)	.23
Conduct problems (.52), Mean (SD)	1.69 (1.50)	1.67 (1.46)	-0.01 (-0.31 - 0.29)		.95
- at risk score, n (%)	18 (13)	16 (11)		1.14 (0.63 - 2.10)	.71
Hyperactivity (.75), Mean (SD)	3.30 (2.33)	3.08 (2.19)[c]	0.23 (-0.23 - 0.68)		.41
- at risk score, n (%)	13 (9)	10 (7)[c]		1.32 (0.65 - 2.69)	.52
Peer problems (.67), Mean (SD)	1.51 (1.91)[b]	1.15 (1.49)	0.38 (0.03 - 0.72)		.07
- at risk score, n (%)	18 (13)[b]	11 (8)		1.75 (0.90 - 3.39)	.17
Prosocial behavior (.67), Mean (SD)	8.10 (1.67	8.03 (1.81)	0.13 (-0.22 - 0.49)		.53
- at risk score, n (%)	4 (3)[b]	6 (4)		0.66 (0.23 - 1.93)	.53
Total difficulties (.78), Mean (SD)	8.59 (5.29)[b]	7.56 (4.57)[c]	1.03 (0.02 - 2.05)		.09
- at risk score, n (%)	10 (7)[b]	4 (3)		2.64 (0.98 - 7.10)	.11

[a] Score range 0-10 for 'Emotional problems', 'Conduct problems', 'Hyperactivity', 'Peer problems', higher scores indicating more behavioral problems. Score range 0-10 for 'Prosocial behavior', lower scores indicating more behavioral problems. Score range 0-40 for 'Total difficulties', higher scores indicating more behavioral problems. The category 'at risk score' corresponds to the top 10% abnormal scores of each scale, based on a large UK community sample.[23] [b] *n* = 139, [c] *n* = 138.

© 2022 Picotti E et al. *JAMA Network Open.*

eTable 2. Comparison of Parent-Reported HRQoL According to the KIDSCREEN-27 Between Groups at Age 5 Years Restricted to Children Who Completed the Allocated Treatment

**KIDSCREEN 27 Questionnaire Dimensions[a] (0-100 range)** ***(Cronbach's Alpha)***	**rhEpo N = 139**	**Placebo N = 140**	**Mean Difference (95% CI)**	***P*-value**
Physical Well-Being (.79), Mean (SD)	74.82 (12.46)[b]	77.20 (12.61)	-2.57 (-5.19 - 0.05)	.11
Psychological Well-Being (.73), Mean (SD)	85.69 (8.83)[b]	86.54 (9.08)[e]	-0.52 (-2.38 - 1.33)	.64
Autonomy and Parent Relation (.63), Mean (SD)	83.46 (9.14)[c]	82.70 (11.32)[f]	1.49 (-0.78 - 3.76)	.28
Social Support and Peers (.78), Mean (SD)	73.70 (15.38)[d]	75.71 (13.65)[d]	-1.92 (-4.98 - 1.14)	.30
School Environment (.81), Mean (SD)	85.97 (12.95)[d]	86.98 (12.04)[g]	-1.19 (-3.80 - 1.42)	.45

[a] Raw scores for all items in each dimension were summed and transformed in a 0-100 continuum, with higher values indicating better HRQoL. [b] *n* = 138, [c] *n* = 116, [d] *n* = 135; [e] *n*= 139; [f] *n* = 114; [g] *n* = 132.

© 2022 Picotti E et al. *JAMA Network Open.*
