# Supplementary material for: Effect of Early High-Dose Recombinant Human Erythropoietin on Behavior and Quality of Life in Children Aged 5 Years Born Very Preterm: Secondary Analysis of a Randomized Clinical Trial
Source: JAMA Netw Open. 2022 Dec 7;5(12):e2245499. doi: 10.1001/jamanetworkopen.2022.45499 (PMC9856490; doi:10.1001/jamanetworkopen.2022.45499)
Supplement: Supplement 3. — The Swiss EPO Neuroprotection Trial Group [file jamanetwopen-e2245499-s003.pdf]

Supplemental Online Content: Nonauthor **Collaborators of the The Swiss EPO Neuroprotection Trial Group**
*First name, last name, and suffix (if applicable) are required and will appear in PubMed.

*Group Name(s): Swiss EPO Neuroprotection Trial Group

***First Name and Middle Initial(s)**	***Last Name**	***Suffix (eg, Jr, III)**	Academic Degrees	Institution	Location (city, state/province, country)	Role or Contribution, eg, chair, principal investigator	Group (if more than 1 Group listed in the byline) and/or Subgroup (eg, Steering Committee)
Georg	Zellinger		MD	Neonatal Unit, Dept of Paediatrics, Ca	Aarau, Switzerland	local investigator	
Sylviane	Pasquier		MD	Neonatal Unit, Dept. of Paediatrics, C	Aarau, Switzerland	local investigator	
Andrea	Capone		MD	Department of Neuropaediatrics, Car	Aarau, Switzerland	local investigator	
Christoph	Bührer		MD Prof	Department of Neonatology, Univers	Basel, Switzerland	local investigator	
René	Glanzmann		MD	Department of Neonatology, Univers	Basel, Switzerland	local investigator	
Sven	Schulzke		MD Prof	Department of Neonatology, Univers	Basel, Switzerland	local investigator	
Peter	Weber		MD	Department of Neuropaediatrics and	Basel, Switzerland	local investigator	
Brigitte	Scharrer		MD	Paediatric and Neonatal Intensive Ca	Chur, Switzerland	local investigator	
Walter	Bär		MD	Paediatric and Neonatal Intensive Ca	Chur, Switzerland	local investigator	
Elmar	Keller		MD	Department of Neuropaediatrics, Car	Chur, Switzerland	local investigator	
Christa	Killer		MD	Department of Neuropaediatrics, Car	Chur, Switzerland	local investigator	
Riccardo	Pfister		MD Prof	Neonatology and Pediatric Intensive	Geneva, Switzerland	local investigator	
Karin	Krämer		MD	Neonatology and Pediatric Intensive	Geneva, Switzerland	local investigator	
Petra S	Hüppi		MD Prof	Division of Development and Growth	Geneva, Switzerland	local investigator	
Cristina	Borradori-Tolsa		MD	Division of Development and Growth	Geneva, Switzerland	local investigator	
Beatrix	Latal		MD Prof	Child Development Centre, University	Zurich, Switzerland	local investigator	
Hans Ulrich	Bucher		MD Prof	Department of Neonatology, Univers	Zurich, Switzerland	local investigator	
Jean-Claude	Fauchère		MD Prof	Department of Neonatology, Univers	Zurich, Switzerland	Principal investigator	
Sven	Welmann		MD Prof	Department of Neonatology, Univers	Zurich, Switzerland	Principal investigator	
Brigitte	Koller		MSc	Department of Neonatology, Univers	Zurich, Switzerland	local investigator	
Giancarlo	Natalucci		MD Prof	Department of Neonatology, Univers	Zurich, Switzerland	local investigator	
