# Supplementary material for: Effect of Early High-Dose Recombinant Human Erythropoietin on Behavior and Quality of Life in Children Aged 5 Years Born Very Preterm: Secondary Analysis of a Randomized Clinical Trial
Source: JAMA Netw Open. 2022 Dec 7;5(12):e2245499. doi: 10.1001/jamanetworkopen.2022.45499 (PMC9856490; doi:10.1001/jamanetworkopen.2022.45499)
Supplement: Supplement 4. — Data Sharing Statement [file jamanetwopen-e2245499-s004.pdf]

Data Sharing Statement

Picotti. Effect of Early High-Dose Recombinant Human Erythropoietin on Behavior and Quality of Life in Children Aged 5 Years Born Very Preterm. *JAMA Netw Open*. Published December 07, 2022. doi:10.1001/jamanetworkopen.2022.45499

Data
Data available: Yes
Data types: Deidentified participant data, Data dictionary
How to access data: A de-identified data set will be provided upon approval of formal request from qualified scientific and/or medical researchers at the study's steering committee. The request shall be sent to Prof. Jean-Claude Fauchère (e-mail: jean.claude.fauchère@usz.ch). Data elements will include variables needed to reproduce our analysis. We will consider sharing additional elements on a case by case basis after approval of a proposal. De-identified data will be shared by means of a data transfer tool in accordance with the ethical and scientific quality standard of Good Clinical Practices. When available: beginning date: Immediately following publication; duration: 5 years after publication.
When available: With publication

Supporting Documents
Document types: Statistical/analytic code, Informed consent form, Other (please specify)
Additional Information: Study protocol.
How to access documents: Statistical/analytic code, informed consent form, and study protocol (already published). How to access documents: the supporting documents will be available after approval of potential requests made to jean.claude.fauchère@usz.ch. These documents will be shared by means of a data transfer tool in accordance with the ethical and scientific quality standard of Good Clinical Practices. When available: beginning date: Immediately following publication; duration: 5 years after publication.
When available: With publication

Additional Information
Who can access the data: Data will be available to qualified scientific and/or medical researchers whose proposed use of the data has been approved by the study's steering committee.
Types of analyses: Data will be made available only for specified research purpose in accordance with the ethical and scientific quality standard of Good Clinical Practices.
Mechanisms of data availability: De-identified data will be shared by means of a data transfer tool in accordance with the ethical and scientific quality standard of Good Clinical Practices.
Any additional restrictions: N/A.
